# Alcohol-induced placental vasculopathy reprograms the placental-fetal cardiovascular axis: angiogenic mechanisms linking maternal alcohol exposure to developmental cardiovascular programming

**DOI:** 10.3389/fphys.2026.1917947

**Published:** 2026-10-12

**Authors:** Elisa Cebral

**Affiliations:** Laboratorio de Reproducción y Fisiología Materno-Embrionaria, Departamento de Biodiversidad y Biología Experimental (DBBE), Instituto de Biodiversidad y Biología Experimental y Aplicada (IBBEA), Consejo Nacional de Investigaciones Científicas y Tecnológicas (CONICET), Facultad de Ciencias Exactas y Naturales, Universidad de Buenos Aires, Buenos Aires, Argentina

**Keywords:** cardiovascular programming, placenta, heart disease, placentopathy, maternal alcohol, early gestation, angiogenesis, feto-maternal remodeling

## Abstract

Among prenatal insults, maternal alcohol consumption has emerged as an important determinant of fetal cardiovascular development and long-term cardiovascular disease susceptibility. Growing evidence indicates that the placenta is a central biological mediator linking maternal alcohol exposure with abnormal fetal cardiac development. This review integrates current experimental and clinical findings on the developmental relationship between placental vascularization and fetal cardiogenesis, highlighting the placental-embryofetal cardiovascular axis as a conceptual framework for alcohol-induced cardiopathy programming. We examine evidence that prenatal alcohol exposure disrupts placental vascular development through impaired decidual spiral artery angiogenesis and remodeling and altered trophoblast differentiation and invasion, and labyrinthine vascularization. Experimental studies suggest that oxidative stress and hypoxia perturb interconnected angiogenic vascular endothelial growth factor (VEGF)-nitric oxide synthases (NOS)-matrix metalloproteinases (MMPs) pathways, and related redox-sensitive mediators, ultimately compromising placental perfusion and fetoplacental hemodynamics. We further discuss that placental dysfunction may alter the molecular, metabolic, endocrine, oxygen-dependent, and hemodynamic signals regulating fetal cardiac development, potentially affecting myocardial morphogenesis, cardiac remodeling, coronary vascularization, septation, valvulogenesis, and cardiovascular maturation during critical developmental windows. Collectively, available evidence supports a model in which disruption of placental angiogenic homeostasis may represent a key intermediary linking maternal alcohol exposure with fetal cardiovascular programming. This perspective integrates direct embryofetal effects with indirect placental-mediated mechanisms and highlights the need to clarify their relative contributions across developmental windows. Improved understanding of the placental-embryofetal cardiovascular axis may facilitate identification of early biomarkers and preventive or therapeutic strategies to reduce the lifelong cardiovascular consequences of prenatal alcohol exposure.

## Introduction

Throughout embryo-fetal development, proper cardiogenesis and fetal circulatory adaptation critically depends on a tightly regulated intrauterine environment, changes in oxygen availability, nutrient supply, endocrine signaling, and hemodynamic conditions (Burton and Fowden, 2015).Following establishment of the maternal-fetal circulation, the cardiogenesis become functionally coupled with placental development, by which a cardio-placental axis emerges as a highly coordinated physiological and molecular unit, finely regulated by angiogenic pathways, such as one of the most relevant, the vascular endothelial growth factor (VEGF) system. Because early cardiogenesis and placentation occur within discrete critical windows of vulnerability, adverse maternal exposures and environmental insults that disturb uteroplacental vascularization and placental function, may impair cardiogenesis, fetal vascular maturation and cardiovascular adaptation, increasing the susceptibility to cardiovascular disease later in life (Tain and Hsu, 2017).

In this context, the placenta has emerged as a central regulator of cardiovascular developmental programming. Beyond its classical functions in nutrient and gas exchange, it acts as a dynamic maternal-fetal interface that integrates immune tolerance, endocrine communication, angiogenic signaling, metabolic homeostasis, oxygen sensing, and hemodynamic regulation, thereby providing the physiological environment required for normal embryofetal growth and cardiovascular development (Guttmacher et al., 2014; Burton and Fowden, 2015; Sferruzzi-Perri and Camm, 2016; Martín-Estal et al., 2019). Within the *Developmental Origins of Health and Disease* (DOHaD) framework, accumulating experimental and clinical evidence indicates that disturbances of placental development and function permanently influence cardiovascular health through interconnected epigenetic, oxidative, inflammatory, endocrine, metabolic, vascular, and hemodynamic mechanisms that shape fetal developmental trajectories (McMillen and Robinson, 2005; Hanson and Gluckman, 2008; Tain and Hsu, 2017; Lapehn and Paquette, 2022). In particular, impaired maternal angiogenesis and uteroplacental vascular dysfunction promote placental malperfusion, placental insufficiency, hypertensive disorders of pregnancy, and preterm birth (Reynolds et al., 2006; Gyselaers, 2023), conditions that are strongly associated with fetal growth restriction, perinatal morbidity and mortality, and an increased susceptibility to hypertension, metabolic syndrome, insulin resistance, type 2 diabetes mellitus, and cardiovascular disease later in life (McMillen and Robinson, 2005; Hanson and Gluckman, 2008; Sharma et al., 2016; Lacko et al., 2017; Pérez-Garcia et al., 2018; Woods et al., 2018). These evidences have led to the emerging concept of the *placenta-embryofetal cardiovascular axis*, whereby the placenta is viewed not as an isolated gestational organ but as a central biological regulator that integrates angiogenic, metabolic, endocrine, and hemodynamic signals governing embryofetal cardiogenesis. Consequently, disruption of placental vascular homeostasis during critical developmental windows may represent a key mechanistic intermediary linking adverse intrauterine exposures to lifelong cardiovascular dysfunction.

Within this conceptual framework, prenatal alcohol exposure has emerged as one of the most prevalent, preventable, and biologically relevant developmental insults. Both experimental and clinical evidence indicate that maternal alcohol consumption simultaneously disrupts placental vascular development at fetal-maternal interface, embryonic cardiac morphogenesis and fetal cardiovascular homeostasis, making it a unique model for investigating how placental dysfunction contributes to lifelong cardiovascular disease susceptibility (Turcotte et al., 2002; Gundogan et al., 2013; Tai et al., 2017; Popova et al., 2023).

Despite widespread public health recommendations advocating complete abstinence during pregnancy, alcohol consumption among women of reproductive age remains highly prevalent and continues to increase in many countries (Popova et al., 2017) including Argentina (López et al., 2015). In the United States, approximately 40% of pregnant women report alcohol consumption during pregnancy, whereas 3-5% continue heavy drinking throughout gestation (Heller and Burd, 2013). Similarly, studies from several Latin American countries have reported particularly high rates of alcohol use during pregnancy, with binge drinking representing a frequent pattern of consumption. In Argentina, a population-based study conducted in Santa Fe Province showed that 75.2% of women consumed alcohol at least once during pregnancy and 15.1% reported at least one binge drinking episode (López et al., 2015). Of particular developmental relevance, more than half of women consume moderate quantities of alcohol during early stages of gestation (around human 4–6 weeks after conception) before pregnancy recognition, when embryogenesis is already underway (Colvin et al., 2007; Heller and Burd, 2013). Consequently, many embryos are exposed to moderate or episodic alcohol intake during the peri-implantation period and early organogenesis, encompassing the critical windows in which placentation, embryonic vasculogenesis, and cardiogenesis are initiated. This exposure pattern, commonly referred to as **“**perigestational alcohol consumption” (PAC), represents one of the most prevalent forms of prenatal alcohol exposure and includes alcohol intake occurring before conception and/or during the earliest stages of pregnancy, often before pregnancy is clinically recognized (Muggli et al., 2016; Cebral et al., 2007, Cebral et al., 2011; Coll et al., 2011, 2017; Pérez-Tito et al., 2014).

Maternal alcohol consumption during pregnancy is the leading preventable cause of *Fetal Alcohol Spectrum Disorders* (FASD), affecting approximately 30-40% of children born to mothers who consume alcohol during gestation and with an estimated global prevalence of 22.8 per 1,000 live births (Bosco and Diaz, 2012; Popova et al., 2017). Traditionally, research on fetal alcohol spectrum disorders (FASD) has primarily focused on the developing central nervous system, and the syndrome is currently recognized as a complex developmental disorder characterized by neurobehavioral impairment, craniofacial dysmorphology, fetal growth restriction, congenital malformations, metabolic disturbances, and persistent cognitive dysfunction (Hoyme et al., 2016; Roozen et al., 2016; Popova et al., 2017, 2023; Gursu et al., 2026). In contrast, the cardiovascular consequences of prenatal alcohol exposure have received comparatively less attention, despite growing clinical and experimental evidence demonstrating an increased incidence of congenital heart defects, vascular abnormalities, impaired cardiac function, and enhanced susceptibility to cardiovascular disease later in life (Tain and Hsu, 2017; Popova et al., 2017, 2023). This emerging evidence suggests that cardiovascular dysfunction represents an underrecognized component of FASD pathophysiology and highlights the need to better understand the developmental mechanisms linking prenatal alcohol exposure to altered cardiovascular outcomes.

Although the mechanisms underlying FASD-associated cardiovascular abnormalities remain incompletely understood, their developmental origin is likely multifactorial and depends on the dose, pattern, timing, and duration of maternal alcohol exposure. Experimental studies have consistently shown that alcohol exposure during early organogenesis induces neural tube dysmorphogenesis, growth delay, impaired cellular differentiation and adhesion, oxidative stress, disruption of vasoactive signaling, changes that are compatible with the structural abnormalities observed in FASD (Cebral et al., 2007; Coll et al., 2011, 2017; Gualdoni et al., 2022a). However, whether similar developmental mechanisms contribute to embryofetal cardiogenesis remains largely unresolved. Increasing evidence suggests that placental vascular injury may represent an additional and previously underrecognized mechanism linking maternal alcohol exposure to abnormal cardiac development, thereby extending the pathogenic paradigm of FASD beyond the direct embryotoxic effects of ethanol (Gupta et al., 2016).

Maternal alcohol consumption disrupts placental morphogenesis and vascular function and has been associated with a spectrum of pregnancy complications collectively referred to as the *alcohol-related placental-associated syndrome* (APS) (Salihu et al., 2011; Burton et al., 2016). Building upon this concept, we propose the term *alcohol-induced vascular placentopathy* to emphasize the central role of placental vascular dysfunction as an integrated pathological process involving impaired angiogenesis, spiral artery remodeling, trophoblast dysfunction, oxidative stress, inflammatory activation, and abnormal fetoplacental hemodynamics. These alterations contribute to miscarriage, placental insufficiency, preeclampsia, placenta previa, placenta accreta, placental hemorrhage, fetal growth restriction, and adverse fetal outcomes (Gundogan et al., 2010; Avalos et al., 2014; Carter et al., 2016; Tai et al., 2017; Ohira et al., 2019; Odendaal et al., 2020). Although the molecular basis of this vasculopathy remains incompletely characterized (Orzabal et al., 2019), experimental mouse models have begun to demonstrate that alcohol disrupts placental angiogenic signaling, nitric oxide homeostasis, extracellular matrix remodeling, and inflammatory pathways, thereby providing mechanistic support for this concept (Ventureira et al., 2019; Gualdoni et al., 2021).

Because ethanol readily crosses the placental barrier, prenatal alcohol exposure may affect fetal development through both direct embryotoxic effects and indirect mechanisms secondary to placental dysfunction. Indeed, abnormal placental development has been associated with fetal growth restriction, congenital malformations, obesity, metabolic syndrome, and cardiovascular disease later in life (Bada et al., 2005; Burd et al., 2007; Gundogan et al., 2008, 2015; Patra et al., 2011; Bosco and Diaz, 2012; Zhu et al., 2015; Linask and Han, 2016; Davis-Anderson et al., 2017; Tai et al., 2017; Ohira et al., 2019). Nevertheless, most studies at present have examined embryonic and/or fetal defects and placental abnormalities as separate developmental processes. Whether alcohol-induced placental vasculopathy actively disrupts the integrated placenta-embryofetal cardiovascular axis during critical windows of development, thereby contributing to lifelong cardiovascular programming, remains largely unknown. This unresolved question provides the conceptual basis for the present review and aligns developmental teratology with the DOHaD paradigm (Barker, 1998; Barker et al., 2010) by positioning placental vascular dysfunction as a potential mechanistic intermediary between prenatal alcohol exposure and adult cardiovascular disease.

Accordingly, this review integrates current experimental and clinical evidence supporting the concept that maternal alcohol consumption disrupts embryofetal cardiovascular development not only through the direct embryotoxic effects of ethanol, but also through alcohol-induced vascular placentopathy, by compromising placental angiogenesis, vascular remodeling, and fetoplacental hemodynamic homeostasis. Particular emphasis is placed on the placenta, as a central regulator of embryofetal cardiogenesis, by reviewing how maternal alcohol exposure alters the coordinated cellular and molecular processes governing placental vascular development, including decidual angiogenesis, trophoblast differentiation and invasion and remodeling, labyrinth formation, and the establishment of the fetoplacental circulation. It is examined the contribution of oxidative stress (OS) and the interconnected VEGF-matrix metalloproteinase (MMP)-nitric oxide synthase (NOS) signaling network as key mechanisms underlying alcohol-induced placental vascular dysfunction. Finally, it is proposed that disruption of the integrated placenta-embryofetal cardiovascular axis constitutes a unifying mechanistic framework through which placental vascular injury contributes to fetal cardiovascular programming and increases lifelong susceptibility to cardiovascular disease. A better understanding of this placental-cardiac axis may facilitate the identification of early biomarkers, preventive strategies, and therapeutic targets aimed at reducing the long-term cardiovascular consequences of prenatal alcohol exposure.

## Developmental cardiogenesis and angiogenic mechanisms underlying fetal cardiovascular programming

The heart is the first functional organ to develop during embryogenesis, and its morphogenesis begins remarkably early in gestation through tightly coordinated angiogenic and morphogenetic events regulated by the VEGF signaling system. Because cardiogenesis proceeds through discrete developmental stages, each characterized by specific molecular and cellular processes, the embryofetal cardiovascular system exhibits distinct windows of vulnerability whose susceptibility to environmental insults depends on both developmental timing and the progressive establishment of placental-embryofetal integration. Defining this temporal sequence is therefore essential for understanding when maternal alcohol exposure may disrupt cardiac development and initiate long-term cardiovascular programming. Owing to the rapid advancement of transgenic technologies, the mouse has become the principal experimental model for investigating cardiovascular development (Kaufman and Bard, 1999). Throughout this review, cardiac development is described according to embryonic day (E), corresponding to the Theiler developmental stages (TS) (Kaufman and Bard, 1999), and gestational day (GD), with GD1 (=E1) defined by the presence of the vaginal plug (**Figure 1**).

**Figure 1:**
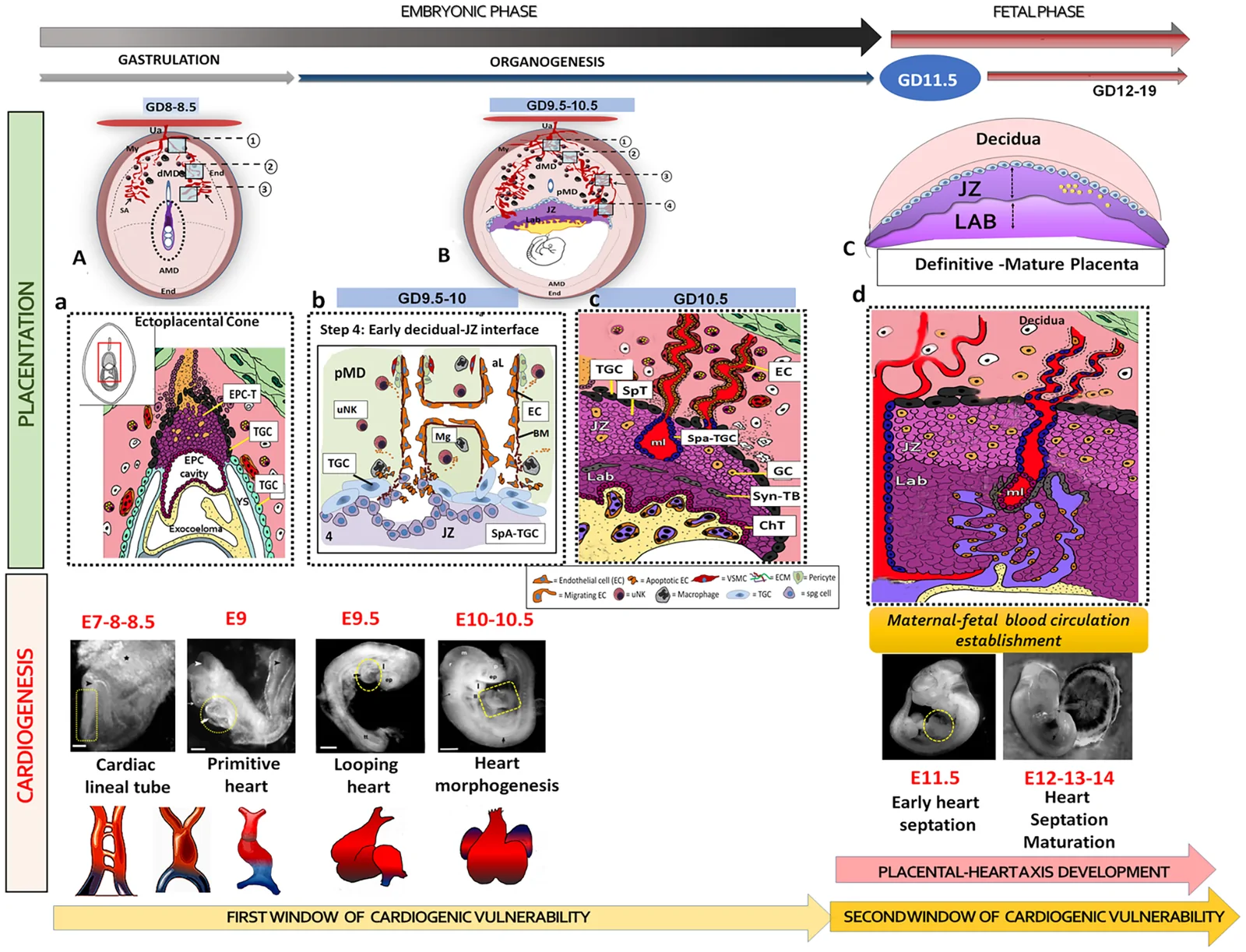
Development and establishment of placental-fetal cardiovascular axis during mouse gestation. Mouse gestation comprises two major developmental periods following implantation: the embryonic phase, including gastrulation (gestational day (GD) 7-8.5) and organogenesis (GD9-11.5), and the fetal phase (GD11.5-19) when definitive placenta is established (GD11.5), and becomes fully functional and growths up to term (GD19). Placental vascular development follows a coordinated spatiotemporal sequences. At GD8-8.5 **(A)**, vascularization of mesometrial decidua (MD) involves progresively angiogenesis and remodeling of maternal spiral arteries (SA) (A, steps 1-3). At GD9.5-10.5 **(B)**, partially remodeled-SAs reach the decidual-junctional zone (JZ) interface, and maternal endothelial cells (EC) is replaced and completed remodeled by invasive trophoblast giant cells (TGC and SpA-TGC) (B, step 4,b), in cooperation with uterine natural killer (uNK) cells and decidual macrophages (Mg) (detailed step 4). In parallel, the ectoplacental cone (EPC) **(a)** develops in the implantation site of GD8-8.5, by trophoblast proliferation and differentiation, to gives rise the fetal placental compartments, including the junctional zone (JZ) and the labyrinth (Lab) at GD10-10.5 **(B, c)**. In Lab, fetal vessels (fv), derived from allantoic vasculogenesis, establish the fetal placental circulation **(B, c)**. Around GD11.5, when the fetal phase begins, junctional maternal sinousoids (ml), linned by trophoblastic cells, reaches the labyrinth, where trophoblast syncytialization (Syn-TB) progresses to form the hemoplacental barrier that separates maternal and fetal blood (GD11.5) **(C, d)**. At this stage, the development of definitive placenta marks the onset of functional placental-fetal cardiovascular axis and the second window of vulnerability for cardiogenesis. In parallel with placentation, embryofetal development proceeds through a sequences of cardiogenic events during gastrulation (E7-8-8.5), organogenic (E9-10.5) and fetal stages (E11.5–12 to term (GD19). CG, glycogen cells; SpT, spongiotrophobastic cells; ChT, chorionic trophoblast cells; Spa-TGC, spiral artery trophoblastic cells.

### Cardiac lineal tube development

Around E7-7.5, the phase of *mesoderm specification* begins, when with gastrulation, embryonic epiblast cells ingress through the primitive streak. Those cells of the future cardiac mesodermal progenitors begin to express specific cardiac markers (Bry+/Flk-1+/Mesp1+), while a different group of precursors differentiates in hemangioblast cells that express Bry+/Flk-1+ (Brade et al., 2013; Meilhac et al., 2014).

Immediatelly, at E8 (TS10), the splanchnic mesodermal cardiac cells differentiate into the first cardiac field and the second cardiac field (Van Vliet et al., 2012; Kloesel et al., 2016), to form anteriorly and bilaterally the *cardiogenic plate.* Predominantly from the first heart field of this cardiogenic plate, and under their BMP and FGF signalings, subjacent endothelial cells differentiate and aggregate to form right and left *endocardial heart tubes* on either embryonic midline side (E8.5, TS11) (Kaufman and Bard, 1999; Harris and Black, 2010; Varner and Taber, 2012; Van Vliet et al., 2012; Meilhac et al., 2014; Kloesel et al., 2016; Kidokoro et al., 2018; Brade et al., 2013).

By E9 (TS12), the bilateral endocardial tubes fuse at the ventral midline to form the *primitive heart tube*, which represents the first functional structure of the embryonic cardiovascular system. As embryonic folding progresses, the linear heart tube becomes surrounded by cardiomyocytes derived predominantly from the first heart field (Moorman et al., 2003) and acquires its characteristic trilaminar organization, consisting of an inner endocardium, an outer myocardium, and the extracellular matrix-rich cardiac jelly. Suspended within the pericardial cavity by the dorsal mesocardium, the primitive heart tube rapidly undergoes anteroposterior regionalization into the bulbus cordis, primitive ventricle, and primitive atrium, which subsequently give rise to the definitive cardiac chambers, and with rostral arterial (aortic sac) and caudal venous poles (right and left sinus horns) (Miquerol and Kelly, 2012; Kelly et al., 2014). At the same time, specialized transition zones become established along the tube and later contribute to the formation of the cardiac septa, valves, and conduction system (Gittenberber-De Groot et al., 2005; Moorman et al., 2003). Although still morphologically immature, the heart already exhibits spontaneous irregular contractions before any autonomic innervation, reflecting the intrinsic contractile properties of differentiating cardiomyocytes.

Between E9.5 (TS13), the primitive heart undergoes rightward *looping*, a critical morphogenetic event that establishes the spatial organization of the future cardiac chambers and brings the major transition zones into close proximity within the inner curvature of the heart (Miquerol and Kelly, 2012). During this process, regional specification of the primitive ventricle, bulbus cordis, and atrium continues, laying the foundation for subsequent chamber septation and valve formation. At this stage, the heart begins coordinated pumping activity, and circulating blood cells become evident, marking the primitive embryo circulation. Although placental circulation has not yet been established at the fetal-maternal interface in the labyrinth, in this stage the embryonic hemodynamic forces are essential for normal cardiac morphogenesis.

### Heart morphogenesis

At E10 (TS14), *heart morphogenesis* accelerates through coordinated structural and functional events that establish the basic architecture of the definitive heart. Within these processes, the following major events could be considered as the most relevant for cardiogenesis during early organogenesis.

#### Cardiac chamber formation

Following completion of cardiac looping, from right to left, the primitive heart undergoes regional spatial organization of bulbus cordis, primitive ventricle (located at a ventral position), the primitive atrium and the right and left horn of the sinus venosus at dorsal position. These sinus horns drain the entire systemic venous return into the atrial chamber, constituting the outflow tract (Kelly et al., 2014). Ventricular ballooning and progressive interventricular septation initiate the formation of the definitive cardiac chambers and the atrioventricular and ventriculoarterial connections (Anderson et al., 2006).

### Myocardial trabeculation and endocardial cushion formation

Rapid myocardial growth, expansion of the cardiac jelly, ventricular trabeculation, and the appearance of the endocardial cushions, which constitute the primordia of the cardiac septa and valves (Gittenberber-De Groot et al., 2005; Kelly et al., 2014; Captur et al., 2016). During trabeculation, myocardial tissue of the inner wall of ventricles, expands toward the cardiac jelly as cardiomyocytes proliférate, delaminate and migrate into the lumen where they form filamentous trabecular structures.Trabeculation, driven by coordinated endocardial-myocardial signaling, increases contractile performance, facilitates oxygen diffusion before coronary circulation develops, generating contractile force required for blood propulsion, and contributing to electrical impulse propagation within the early developing heart (Wagner and Siddiqui, 2007).

### Epicardial development

From E10 onward, proepicardial progenitor cells derived from the mesenchyme of the septum transversum progressively cover the myocardial surface and undergo epithelial-to-mesenchymal transition, giving rise to coronary vascular smooth muscle cells, fibroblasts, and connective tissue that are essential for coronary vessel formation and myocardial maturation (Smits et al., 2017).

### Cardiovascular function

Cardiomyocytes acquire progressively organized sarcomeres and rhythmic contractions become coordinated. Thus the sinus venosus functions as the primary pacemaker of the embryonic heart. As cardiac output increases, blood flow-generated biomechanical forces begin to actively regulate cardiac remodeling, chamber maturation, and vascular development.

Collectively, the high susceptible E10-cardiogenic events, including cardiomyocyte proliferation, ventricular trabeculation, chamber septation, embryonic blood flow, may be parts of the most critical stage window of embryo cardiovascular vulnerability. Importantly, this developmental window temporally overlaps with the onset of labyrinth formation and maternal vascular remodeling at junctional trophoblast interface of the placenta denoting that this early vascular and circulation development of maternal and fetal faces of placenta becomes increasingly coordinated with early cardiac morphogenesis (**Figure 1**). Consequently, this early cardiogenic period is particularly susceptible to adverse intrauterine exposure of maternal alcohol ingestión during early organogenesis.

Building upon the morphogenetic events initiated at E10, by E10.5 (TS15) *(heart remodeling*) cardiac septation and valvulogenesis establish through the remodeling of the endocardial cushions. Localized swellings develop within the atrioventricular canal and the common outflow tract, giving rise to the atrioventricular and outflow tract cushions, which subsequently form the cardiac septa and valves (DeLaughter et al., 2016). Cushion development depends on epithelial-to-mesenchymal transition (EMT) of endocardial cells, induced by myocardial-derived signals including TGF-β2, BMP2, and VEGF-A, allowing mesenchymal cells to invade the extracellular matrix-rich cardiac jelly. The superior and inferior cushions progressively expand and fuse around E12, thereby establishing the structural foundation for definitive cardiac septation and valvular morphogenesis (Wagner and Siddiqui, 2007). Because endocardial cushion formation is critically dependent on coordinated angiogenic and epitelial-mesenchymal signaling, E10.5 represents another highly vulnerable developmental window in which placental vascular dysfunction and altered fetoplacental homeostasis may compromise normal cardiac septation and valve development.

### Early heart septation

By E11.5 (TS16) (*early heart septation*), cardiac morphogenesis enters a stage of complex structural remodeling characterized by interatrial and interventricular septation, subdivision of the primitive outflow tract into the aortic and pulmonary channels, and progressive maturation of the atrioventricular and semilunar valves (Anderson et al., 2003; Savolainen et al., 2009). Septation is achieved through coordinated growth of the muscular septa, separating the right and left atria, together with epithelial-to-mesenchymal transition and fusion of the atrioventricular and conotruncal endocardial cushions, which ultimately establish the definitive separation of the cardiac chambers and great vessels (Kloesel et al., 2016). Thus, the atrioventricular cushions contribute to separation of the atrial and ventricular chambers by formation of the mitral and tricuspid valves, whereas the conotruncal and intercalated cushions participate in division of the outflow tract and formation of the pulmonary and aortic semilunar valves (Savolainen et al., 2009).

Importantly, this developmental stage coincides with the establishment of the definitive chorioallantoic placenta and the functional fetoplacental circulation (**Figures 1C, d**). As maternal blood reaches the placental labyrinth and maternal-fetal exchange becomes fully established, fetal cardiac development becomes increasingly influenced by placental vascular function, oxygen and nutrient delivery, endocrine and angiogenic signaling, and the hemodynamic forces generated by the fetoplacental circulation. Consequently, cardiogenic E11.5 represents the onset of the second developmental window of cardiovascular vulnerability, during which placental vascular dysfunction may profoundly influence cardiac remodeling, septation and coronary vascular development, suggesting that this early fetal cardiogenic stage may contribute to long-term programming of cardiovascular function.

### Heart septation and maturation

Between E12 and E13 (TS17-19) (*late septation phase*), cardiac septation progresses toward completion through continued growth of the muscular interventricular septum and fusion of the atrioventricular and conotruncal endocardial cushions, resulting in definitive separation of the atrial and ventricular chambers (Butcher and Markwald, 2007). During this period, atrial septation is completed by formation of the septum secundum and the foramen ovale, which maintains the physiological right-to-left shunt characteristic of fetal circulation. Concurrently, endocardial cushion mesenchyme progressively differentiates into valve interstitial fibroblasts responsible for extracellular matrix deposition and maturation of the atrioventricular and semilunar valves (Butcher and Markwald, 2007).

From E14.5 onward (*heart maturation*), cardiac morphogenesis gradually reaches completion as the compact myocardium expands, coronary vascularization develops, and the cardiac conduction system, including differentiation of Purkinje fibers, becomes functionally established. During the remainder of gestation, the fetal heart acquires its definitive three-dimensional architecture through progressive structural and functional maturation of the myocardium, valves, and conduction network. Although atrial septation is anatomically completed before birth, functional closure of the foramen ovale occurs after delivery in response to postnatal hemodynamic changes, whereas permanent anatomical closure follows subsequent tissue fusion (Anderson et al., 2003; Savolainen et al., 2009; Kloesel et al., 2016).

In summary (**Figure 1**), cardiogenesis is divided into three conceptual blocks, consisting of stages *E7-E10.5*, when the cardiac program is established, in which disruptions in this tightly coordinated process may result in severe structural cardiac malformations, permanent defects in cardiovascular development and function (Conway et al., 2003). The stages *E11.5-E1*3, defined by establishment of the placenta-fetal cardiac functional axis, definitive septation and beginning of dependence on the fetoplacental circulation; and stages *E14-term*: cardiac maturation. In overall, with the definitive chorioallantoic placenta and the fetoplacental circulation establishment (**Figures 1C, d**), fetal cardiogenesis and maturation becomes increasingly dependent on placental vascular competence, thus regulating fetal heart development through integrated angiogenic, endocrine, metabolic, and hemodynamic signals. Consequently, persistent placental dysfunction during this fetal period may alter myocardial maturation, coronary vascular development, valve remodeling, and cardiac functional adaptation, thereby contributing to cardiovascular programming and increasing susceptibility to cardiovascular disease later in life.

Embryofetal cardiovascular development is a highly dynamic process that depends on the precise temporal and spatial coordination of genetic, molecular, and hemodynamic signals. Among the signaling pathways governing these events, the vascular endothelial growth factor (VEGF) system plays a central role in both placental vascular development and cardiovascular morphogenesis and thereby representing a key molecular regulator of the placenta-fetal cardiovascular axis.

Beyond its canonical function in vasculogenesis and angiogenesis, VEGF signaling orchestrates multiple aspects of cardiac development, including endothelial and endocardial specification, myocardial trabeculation, coronary vessel formation, endocardial cushion maturation, and valvulogenesis, thus coordinating the structural and functional maturation of the embryofetal cardiovascular system (Simons et al., 2016; Zhang et al., 2021; Trimm and Red-Horse, 2023). Accordingly, VEGF is currently recognized as a master regulator of cardiovascular development, integrating vascular patterning, tissue morphogenesis, and embryonic vascular homeostasis (Pérez-Gutiérrez and Ferrara, 2023; Trimm and Red-Horse, 2023).

Importantly, VEGF signaling exhibits highly dynamic and stage-specific patterns of expression throughout embryofetal cardiogenesis, orchestrating successive heart development and morphogenesis during discrete windows of cardiovascular morphogenesis.

During gastrulation and early vasculogenesis (E7-E8.5), VEGF released by the somitic mesoderm and primitive endoderm, in concert with Sonic hedgehog (Shh) and Notch signaling, directs angioblast migration, endothelial specification, and assembly of the primary vascular network, including formation of the dorsal aorta, cardinal veins, and intersomitic vessels, while inhibitory signals from the notochord establish the avascular embryonic midline (Coultas et al., 2005). As cardiogenesis progresses (E9-E10.5), VEGF signaling assumes additional roles in cardiac morphogenesis. At this stage, VEGF is highly expressed in the endocardium and adjacent myocardium of the atrioventricular canal and outflow tract, where it finely regulates endocardial epithelial-to-mesenchymal transition (EMT), endocardial cushion formation, and subsequent septal and valvular morphogenesis. Notably, VEGF activity is tightly dosage-dependent: insufficient signaling impairs mesenchymal proliferation and cushion growth, whereas excessive VEGF expression suppresses EMT, ultimately disrupting valve development (Wagner and Siddiqui, 2007). During subsequent stages (E10.5-E13) of cardiac maturation, VEGF also promotes coronary vasculogenesis by inducing endocardial-derived endothelial cells to invade the developing myocardium and establish the coronary vascular network (Wu et al., 2012).

The temporal regulation underscores the concept that precise VEGF signaling is required throughout cardiogenesis. Disruption during specific developmental windows potentially leads to distinct structural and functional cardiovascular abnormalities. Experimental studies using genetically modified murine models have demonstrated that even modest reductions in VEGF expression profoundly disrupt cardiovascular morphogenesis and vascular patterning. Accordingly, VEGF-null embryos, including heterozygous haploinsufficient mice, exhibit defective endothelial differentiation, impaired vascular organization, cardiac malformations, and abnormal hematopoietic development, leading to embryonic lethality around E9.5 (Carmeliet et al., 1996; Ferrara et al., 1996).The importance of maintaining appropriate VEGF signaling throughout cardiogenesis is further underscored by human genetic studies demonstrating associations between reduced VEGF expression and several congenital heart defects, including DiGeorge syndrome, Tetralogy of Fallot, septal defects, and valvular malformations (Van den Akker et al., 2012).

VEGF signaling is orchestrated through two functionally complementary receptors that together determine the magnitude, timing, and spatial distribution of VEGF activity during embryonic cardiovascular development. The KDR receptor functions as the principal signaling receptor, promoting endothelial lineage specification, endothelial cell proliferation, migration, survival, and vascular assembly, thereby driving formation of blood islands and primary vascular plexuses in embryos (Simons et al., 2016; Peach et al., 2018; Apte et al., 2019; Trimm and Red-Horse, 2023). During gastrulation, KDR-expressing mesodermal progenitors migrate through the primitive streak to populate both embryonic and extraembryonic mesoderm, where KDR-positive hemangioblasts generate the endothelial and hematopoietic lineages that establish the primitive vascular network (Coultas et al., 2005; Haigh, 2008). Beyond vasculogenesis, KDR signaling also regulates endothelial mechanosensing and angiogenic remodeling during cardiovascular morphogenesis (Park et al., 2022). In contrast, Flt-1 primarily acts as a modulatory receptor, limiting VEGF bioavailability and establishing the spatiotemporal signaling gradients required for coordinated vascular patterning and cardiac morphogenesis (Peach et al., 2018; Pérez-Gutiérrez and Ferrara, 2023). The complementary functions of both receptors are clearly illustrated by genetic mouse models: whereas KDR deficiency prevents vasculogenesis and causes embryonic lethality between E8.5 and E9.5 owing to failure of blood island and primitive vascular plexus formation (Shalaby et al., 1995), Flt-1-null embryos die at a similar developmental stage because uncontrolled VEGF signaling induces vascular hyperplasia and severe vascular dysfunction (Fong et al., 1995). Moreover, restoration of embryonic development by expression of a kinase-deficient Flt-1 receptor demonstrated that the essential developmental role of Flt-1 is not intracellular signal transduction, but rather the regulation of VEGF availability and signaling gradients necessary for normal cardiovascular morphogenesis (Hiratsuka et al., 1998).

Collectively, these findings indicate that embryofetal cardiovascular development is governed by a highly coordinated developmental signaling landscape that depends not only on adequate VEGF expression but also on the dynamic balance between KDR-mediated pathways and Flt-1 expression and mediated-ligand sequestration. Moreover, VEGF operates within an integrated angiogenic network involving also both enzymes NOS and MMPs, and the hypoxia-inducible factor-1 (HIF-1) and redox-sensitive pathways. However, beyond its central role of VEGF, normal embryofetal cardiovascular morphogenesis and homeostasis depends on oxygen availability, placental hemodynamic forces, metabolic and nutrient supply, endocrine communication, and paracrine signals originating from the placenta during critical developmental window. Consequently, normal placental integrated angiogenesis-vascularization emerges as key regulator of embryofetal cardiogenesis and heart developmental programming later in life.

## Placental vascularization as a central mediator of cardiovascular programming

Normal embryofetal cardiovascular development is highly dependent on the intrauterine environment, which is shaped by the dynamic interplay of oxygen and nutrient availability, endocrine and immune signaling, metabolic homeostasis, and hemodynamic conditions at the maternal-fetal interface (Guttmacher et al., 2014; Martín-Estal et al., 2019). Within this regulatory framework, the placenta acts as the central organ coordinating maternal-fetal communication through endocrine, immune, metabolic, and vascular functions (Guttmacher et al., 2014; Sferruzzi-Perri and Camm, 2016; Martín-Estal et al., 2019). Accordingly, normal placental vascular development, achieved through tightly regulated angiogenesis and vascular remodeling, is essential for maintaining fetal homeostasis and ensuring appropriate cardiovascular development (Reynolds et al., 2006; Camm et al., 2018; Creeth and John, 2020). Conversely, impaired fetomaternal angiogenesis and placental vascular dysfunction may lead to placental malperfusion, vascular placentopathy, and gestational complications (Reynolds et al., 2006; Gyselaers, 2023). By altering oxygen and nutrient delivery, endocrine signaling, and fetal hemodynamics during critical developmental windows, these abnormalities interfere with cardiogenesis, vascular maturation, and fetal circulatory adaptation, thereby increasing susceptibility to congenital cardiovascular abnormalities and long-term cardiovascular programming (Corstius et al., 2005; McMillen and Robinson, 2005; Black et al., 2012). Understanding the cellular and molecular mechanisms governing normal placentation, angiogenesis, and placental vascularization provides the essential biological framework for interpreting how prenatal alcohol exposure disrupts the placental-embryofetal cardiovascular axis.

During hemochorial placentation, establishment of the maternal-fetal vascular interface depends on tightly coordinated processes of vasculogenesis, angiogenesis, and vascular remodeling that ensure efficient maternal-fetal exchange. These processes generate a low-resistance, high-capacitance uteroplacental circulation capable of delivering maternal blood at high volume and low velocity. The coordinated development of decidual angiogenesis, spiral artery remodeling, trophoblast differentiation and invasion, and placental vascularization, provide the physiological bases for establishment of the placenta-fetal cardiovascular axis, through which prenatal alcohol exposure may disrupt placental homeostasis and impair cardiovascular development.

The mouse is the most widely used experimental model for investigating the cellular and molecular mechanisms governing placental vascular development and its relationship with embryofetal cardiovascular programming. Despite species-specific differences, both mouse and human placentas are hemochorial, allowing direct contact between maternal blood and trophoblast cells and sharing the fundamental biological processes that regulate trophoblast differentiation and invasion, maternal spiral artery remodeling, and establishment of the maternal-fetal interface. The principal anatomical difference lies in placental organization, with a labyrinthine placenta in mice and a villous placenta in humans. Nevertheless, these conserved developmental mechanisms make the mouse a highly valuable model for investigating placental angiogenesis and vascular remodeling under both physiological and pathological conditions.

Because placentation and cardiogenesis occur according to species-specific developmental timelines, the translational interpretation of mouse studies requires careful alignment of experimental stages with their human gestational equivalents. This comparative developmental approach is particularly important when evaluating the effects of prenatal alcohol exposure during critical windows of placental and cardiovascular development.

The mouse hemochorial placenta consists of the decidua, as the maternal face, the junctional zone (JZ) and the labyrinth (Lab), as components of fetal face of placenta. During gestational days (GD) of development, the interconnected decidual and trophoblastic tissues play essential roles in the formation of definitive placenta from GD 11.5. To provide insight into placental vascularization, next the main events of maternal and fetal angiogenesis are summarized in the mouse model.

### Vascular morphogenesis of maternal face during mouse placentation

Development of the maternal placental vasculature is a fundamental prior event of placentation that transforms the uterine circulation into a low-resistance, high-capacitance vascular bed capable of supporting the increasing metabolic demands of pregnancy (Mu and Adamson, 2006; Brosens et al., 2019). This process involves extensive decidual angiogenesis, remodeling of the spiral arteries (SAs), and progressive enlargement and increased compliance of the maternal vascular network, ultimately reducing uteroplacental vascular resistance.

In mice, as in humans (Smith et al., 2009), maternal spiral artery remodeling proceeds through two temporally coordinated phases.The first, a trophoblast-independent phase (approximately GD7-10), is driven primarily by decidual cells and establishes the initial maternal vascular network through angiogenesis and early spiral artery remodeling. The second, a trophoblast-dependent phase (from approximately GD10.5-11.5 onward), involves coordinated interactions between invasive trophoblasts and decidual cells that complete spiral artery remodeling at the decidua-junctional zone interface, thereby establishing the definitive uteroplacental circulation (Cross et al., 2002; Rai and Cross, 2014; Rusidzé et al., 2023).

The *trophoblast-independent phase of maternal vascular* remodeling is driven by decidualization of the endometrium, the first essential event required for normal maternal angiogenesis and spiral artery remodeling (Croy et al., 2003, 2009, 2012; Ramathal et al., 2010). Following implantation, decidualization begins within the antimesometrial endometrium (GD6-6.5), where stromal cells differentiate to form the primary decidual zone. This initially avascular tissue rapidly expands toward the mesometrial region of the implantation site by GD7.5, while the surrounding extracellular matrix undergoes extensive remodeling, including changes in collagen organization, distribution, and fibril thickness (Dey et al., 2004; Wang et al., 2004; Carbone et al., 2006). These structural changes create the microenvironment required for subsequent angiogenesis, spiral artery remodeling, and trophoblast invasion.

At the mesometrial side of early mouse implantation site, radial uterine artery crosses the myometrium, reaches the endometrium, and branches in arteries and arterioles in the distal mesometrial decidua.These unremodeled SAs (**Figure 1A**, step 1) are characterized by an intact typical wall with pericytes and vascular smooth muscle cells (VSMCs) and endothelial cells (ECs), which respond to the hypoxia state and to several EC and decidual stromal cell-secreting angiogenic factors (Sojka et al., 2019; Gualdoni et al., 2023).

Beginning around GD8-8.5, decidual angiogenesis is initiated by *vascular destabilization* (**Figure 1A**, step 2). During this stage, uterine natural killer (uNK) cells and decidual macrophages accumulate around the spiral arteries (SAs) and secrete angiogenic mediators, including VEGF, MMPs and NO. These factors promote degradation of the endothelial basement membrane, extracellular matrix remodeling, detachment of VSMCs and pericytes, and increased vascular permeability, thereby creating the permissive microenvironment required for endothelial activation and migration. This is followed by *angiogenic sprouting* (**Figure 1A**, step 3), characterized by endothelial cell proliferation and migration, together with continued extracellular matrix remodeling and progressive enlargement of the spiral artery lumen. VEGF signaling coordinates endothelial growth, whereas NO contributes to vasodilation and increased vascular permeability, facilitating maternal blood perfusion into the developing decidua.

Concomitantly, the first processes of spiral artery remodeling involves progressive loss of the VSMC layer from SA, a critical event that transforms unremodeled contractile arterioles into large-capacitance, low-resistance vessels capable of supplying the developing placenta. Whereas distal mesometrial spiral arteries (**Figure 1A**, step 1) initially possess a thick smooth muscle coat, this layer progressively disappears from GD8-8.5 onward and is largely absent in the proximal decidua by GD10-11 (**Figure 1B**, step 4) (Zhang et al., 2011; Ventureira et al., 2019). As a consequence, the remodeled spiral arteries become permanently dilated and lose their vasomotor capacity, ensuring high-volume, low-velocity maternal blood flow to the placental implantation site.

At mid-gestation (GD10-10.5) (**Figure 1B**), ramified, dilated, and partially remodeled SAs, forming a dense capillary plexus of maternal blood at the lateral regions of the proximal mesometrial decidua, converge at the JZ to be remodeled completely by the trophoblastic cells and developing maternal sinusoidal lacunae surrounded by junctional trophoblastic cells (**Figures 1B, b**, step 4, and c) (Croy et al., 2013; Cross et al., 2002; Ventureira et al., 2019). In this stage, the process of *tubulogenesis* involves EC and pericyte recruitment and adhesion into new vessels; basement membrane formation; ECM remodeling; EC differentiation and adhesion, and SA branching, elongation, anastomosis, vessel looping, and vasodilatation, yielding to large luminal extensions of maternal decidual vessels (**Figure 1C**, step 4).

The trophoblast-independent phase of maternal vascular remodeling is coordinated by decidual stromal cells together with maternal immune cells, principally uterine natural killer (uNK) cells and decidual macrophages (Ramathal et al., 2010; Croy et al., 2003, 2009, 2012). Distributed throughout the decidual stroma and around spiral arteries, these cell populations establish a highly coordinated immunoangiogenic microenvironment that initiates maternal vascular remodeling before trophoblast invasion (Croy et al., 2011; Chambers et al., 2021).

Through reciprocal interactions, uNK cells and decidual macrophages orchestrate endothelial activation, extracellular matrix remodeling, vascular smooth muscle cell loss, and the progressive transformation of spiral arteries by releasing angiogenic and vasoactive mediators, including VEGF, PlGF, NO, MMP-2 and MMP-9, TGF-β, IFN-γ, and other cytokines (Ashkar et al., 2000; Wang et al., 2003; Croy et al., 2006; Zhang et al., 2011; Lima et al., 2014; Harris et al., 2019; Sojka et al., 2019; Chambers et al., 2021). Collectively, these mediators constitute an integrated angiogenic signaling network that drives decidual angiogenesis and the early stages of spiral artery remodeling.

Disruption of this finely regulated immunoangiogenic balance by oxidative stress or inflammatory stimuli, including those induced by prenatal alcohol exposure, impairs the physiological phenotype of decidual immune cells, compromises spiral artery remodeling, and promotes placental vascular dysfunction (Li et al., 2017; Ventureira et al., 2019). Because VEGF, MMPs, and NO represent central downstream effectors of these cellular interactions, dysregulation of the VEGF system signaling has emerged as a key mechanism linking alcohol-induced oxidative stress with abnormal placental vascular development.

The *trophoblast-dependent phase of maternal vascular* remodeling begins once the chorioallantoic placenta is established (approximately GD10.5), coincident with the formation of labyrinthine vasculature (**Figure 1B, b and c**). During this period, continuous differentiation of trophoblast giant cells (TGCs) and spongiotrophoblast cells within the junctional zone (JZ) gives rise to the invasive trophoblast populations responsible for the final stages of spiral artery remodeling (Watson and Cross, 2005; Hu and Cross, 2010; Rai and Cross, 2014; Woods et al., 2018; Gualdoni et al., 2021). Maternal spiral arteries reach the JZ already partially remodeled following the trophoblast-independent phase, with marked loss of vascular smooth muscle cells. Subsequently, interstitial and endovascular trophoblasts invade the decidua and maternal vessels, where spiral artery-associated trophoblast giant cells (SpA-TGCs) progressively replace the maternal endothelium, establishing a trophoblast-lined pseudoendothelium (Cross et al., 2002; Croy et al., 2012; Rusidzé et al., 2023). This process is facilitated by trophoblast-derived MMPs, which degrade the extracellular matrix and vascular basement membrane, thereby promoting trophoblast migration and completion of spiral artery remodeling (Rossant and Cross, 2001; Adamson et al., 2002; Varberg and Soares, 2021).

The combined actions of invasive trophoblasts, decidual immune cells, and angiogenic mediators ultimately transform the spiral arteries into permanently dilated, low-resistance vessels capable of sustaining the progressive increase *in utero*placental blood flow required for fetal growth. Although endovascular trophoblast invasion is relatively shallow in mice compared with humans, uNK cells and decidual macrophages continue to cooperate with trophoblasts by producing MMPs, nitric oxide, and other angiogenic mediators that complete maternal vascular remodeling (Huhn et al., 2021).

Thus, successful spiral artery remodeling depends on the coordinated interaction between trophoblast differentiation, decidual immune cells, and angiogenic signaling, making this stage particularly vulnerable to environmental insults such as the prenatal alcohol exposure.

### Vascularization of the fetal face during mouse placentation

Placental development involves a well-defined temporal sequence of fetal face morphogenesis in which the transition from histiotrophic chorionvitelline placenta to hemotrophic nutritional one progressively establishes the chorioallantoic placenta. During early murine development, before GD9.5, the embryo is nourished by uterine secretions absorbed through the visceral and parietal yolk sacs. Following chorioallantoic fusion (GD9-9.5), and from mid-gestation (GD11.5), the definitive placenta assumes the exchange of oxygen and nutrients required to sustain rapid fetal growth.

Fetal face of placenta begins with implantation. Immediately, derived from the mural trophectoderm of implanted blastocyst, polyploid primary trophoblast giant cells (TGCs) invades maternal decidua, whereas the polar trophectoderm proliferates to form the extraembryonic ectoderm, and from it becomes differentiation the chrorionic trophoblast to arise the ectoplacental cone (EPC), around GD8-8.5. From EPC periphery, secondary TGCs emerge and acquire invasive phenotype to actively remodel the decidual extracellular matrix and maternal microvasculature (Malassine et al., 2003) (**Figures 1A, a**).

During organogenesis, with chorion trophoblast and allantois attachment and fusion (GD9-9.5) the chorioallantoic placenta is established. After allantoic mesoderm invades the chorionic trophoblast, fetal capillaries develops by vasculogenesis from mesenchyme to giving rise the fetal vascular network of the labyrinth (GD10-10.5) (**Figures 1B, c**) (Watson and Cross, 2005; Hu and Cross, 2010; Rai and Cross, 2014; Woods et al., 2018). Concurrently, with the labyrinthine progressive branching of the fetal capillaries, differentiation of chorionic trophoblasts develops in the syncytiotrophoblastic and sinusoidal-TGC cells that establish the interhemal barrier (GD11-11.5) (**Figures 1C, d**). In mouse, this barrier consists of the fetal endothelium, its basement membrane, two syncytiotrophoblast layers, and a single sinusoidal-TGC layer, this last one located towards the lumen of the blood spaces of maternal sinusoids.The placental interhemal barrier is highly specialized placental structure that closely interdigitated between maternal and fetal circulations, to allow efficient exchange of oxygen, nutrients, hormones, and metabolic products. Consequently, integrity of the interhemal barrier become essential for maintaining uteroplacental perfusion and for supporting fetal cardiovascular development during the second half of gestation.

The other layer of the fetal face of placenta developing in GD10-10.5 (**Figure 1B**) is the junctional zone (JZ), developed by differentiation of EPC-trophoblast progenitors into spongiotrophoblastic cells (SpT) and the parietal TGCs at the interphase with decidua. Immediatelly, in JZ becomes differentiates the glycogen cells (GCs) (Woods et al., 2018) (**Figures 1B, c**). Junctional parietal TGCs and GCs invade and remodel decidual extracellular matrix, while junctional spiral artery-associated TGCs progressively replace the maternal endothelium of spiral arteries that arrive to JZ, transforming them into permanently dilated, low-resistance vessels that ensure adequate maternal blood flow to the developing placenta (Cross et al., 2002; Rai and Cross, 2014).

By GD11.5, with the development of the JZ and the labyrinth, and within it the establishment of the placental interhemal barrier and the maternal-fetal circulation, the definitive placenta is formed (**Figures 1C, d**).

Defective trophoblast invasion, incomplete endothelial replacement, and/or impaired labyrinthine vascularization may constitute major mechanisms of placental vascular dysfunction and provides the biological basis for subsequent fetal potential altered structural and functional cardiovascular programming (Pérez-Garcia et al., 2018).

## Molecular regulation of placental angiogenesis and vascular remodeling

The processes of placental angiogenesis require the regulation of a complex paracrine network of factors of which one of the most important is the system integrated by the VEGF, the MMPs and the nitric oxide synthases (NOS) (Gualdoni et al., 2022b, 2023). Imbalances of these factors may lead to aberrant placental vascular morphogenesis, resulting in early miscarriage, defective placentation and/or increasing risk of pregnancy-related disorders.

VEGF is one of the principal angiogenic regulators orchestrating placental vascular development throughout gestation. During early pregnancy, VEGF is expressed by trophoblast cells, decidual stromal cells, uterine natural killer (uNK) cells, endothelial cells, and VSMCs within the maternal decidua (Hoffmann et al., 2007; Zhang et al., 2011; Li et al., 2014; Ventureira et al., 2019). Its expression is tightly regulated by oxygen tension through activation of hypoxia-inducible factor-1α (HIF-1α), a master transcriptional regulator of cellular adaptation to hypoxia (Maltepe et al., 2005). During the low-oxygen environment, characteristic of early placental development, stabilization of HIF-1α promotes transcription of multiple genes involved in trophoblast proliferation, differentiation, and angiogenesis, thereby supporting establishment of the ectoplacental cone and normal development of the maternal-fetal interface (Takeda et al., 2006). As placental oxygenation progressively increases, HIF-1α levels decline through oxygen-dependent degradation, allowing the transition toward a more differentiated placental phenotype. Conversely, persistent hypoxia associated with oxidative stress maintains HIF-1α activation while promoting the production of anti-angiogenic mediators, ultimately impairing trophoblast differentiation, spiral artery remodeling, and placental angiogenesis (Woods et al., 2018; Pérez-Garcia et al., 2018).

The biological actions of VEGF are mediated through three receptor tyrosine kinases: VEGFR1 (Flt-1), VEGFR2 (KDR/Flk-1), and VEGFR3 (Flt-4) (Kimura and Esumi, 2003). Among these receptors, VEGFR2 is the principal signaling receptor responsible for endothelial proliferation, migration, vascular permeability, and angiogenic remodeling, whereas VEGFR1 primarily modulates VEGF bioavailability by acting as a ligand-binding decoy receptor (Chung and Ferrara, 2011). Activation of the VEGF-VEGFR2 signaling pathway promotes decidualization, trophoblast proliferation and invasion, placental vascular growth, and maternal-fetal nutrient exchange (Reynolds and Redmer, 2001; Haghighi Poodeh et al., 2012; Lara et al., 2018). In pathological pregnancies characterized by placental vascular dysfunction, reduced VEGF expression together with increased circulating soluble Flt-1 (sFlt-1) shifts the angiogenic balance toward an anti-angiogenic state, contributing to endothelial dysfunction, impaired placental perfusion, and defective vascular remodeling (Roberts and Escudero, 2012; Li et al., 2014).

Downstream of VEGFR2 activation, several intracellular signaling pathways regulate the expression of numerous angiogenic mediators, including endothelial nitric oxide synthase (eNOS) and MMPs (Apte et al., 2019). Nitric oxide (NO), generated by eNOS through the oxidation of L-arginine, represents one of the major downstream effectors of VEGF signaling in the placenta. NO promotes vasodilation of the uteroplacental circulation by inducing vascular smooth muscle relaxation, increasing placental blood flow, and inhibiting platelet aggregation and thrombosis, thereby contributing to the maintenance of adequate fetoplacental perfusion throughout gestation (Krause et al., 2011).

Among the principal downstream effectors of VEGF signaling, MMPs constitute a major regulatory system governing extracellular matrix remodeling during placental angiogenesis. MMPs comprise a family of zinc-dependent endopeptidases that includes collagenases, gelatinases, stromelysins, matrilysins, membrane-type MMPs, and other related proteases (Amălinei et al., 2007; Cui et al., 2017; Henriet and Emonard, 2019). Beyond their well-established proteolytic activity, MMPs regulate multiple biological processes essential for placental development, including cell proliferation, migration, differentiation, apoptosis, extracellular matrix turnover, tissue remodeling, and trophoblast survival and invasion (Lemaître and D’Armiento, 2006; Isaka et al., 2003; Agaoglu et al., 2016; Hamutoğlu et al., 2020).

Among this family, MMP-2 (gelatinase A) and MMP-9 (gelatinase B) play particularly important roles throughout implantation and placentation. They participate in endometrial remodeling during embryo implantation (Novaro et al., 2002), decidualization (Fontana et al., 2012), trophoblast invasion (Staun-Ram et al., 2004; Plaks et al., 2013; Gualdoni et al., 2021), endothelial morphogenesis and remodeling of the uteroplacental extracellular matrix required for maternal vascular adaptation (Sternlicht and Werb, 2001). During murine placental development, trophoblast giant cells express both MMP-2 and MMP-9 from early gestation (Alexander et al., 1996; Bany et al., 2000; Bai et al., 2005). As placentation progresses, MMP-9 becomes the predominant gelatinase regulating trophoblast invasion, spiral artery remodeling, and labyrinthine vascularization during mid-gestation (Fontana et al., 2012). In particular, the strong expression of MMP-9 in parietal trophoblast giant cells around gestational day 10 supports its close association with trophoblast differentiation and invasive capacity, whereas its predominant localization within the spongiotrophoblast suggests a key role in VEGF-dependent vascular remodeling of the junctional zone (Gualdoni et al., 2021).

Accumulating evidence indicates that VEGF and MMPs operate as components of a tightly integrated angiogenic network rather than as independent regulatory pathways. VEGF directly stimulates MMP-2 and MMP-9 expression in endothelial cells, whereas reciprocal interactions between these gelatinases and VEGF signaling coordinate trophoblast invasion, extracellular matrix remodeling, endothelial migration, and labyrinth vascular development (Staun-Ram et al., 2004; Heo et al., 2010; Gualdoni et al., 2022b). Our experimental studies evaluating VEGF together with MMP-2 and MMP-9 expression during murine placentation further support the concept that an appropriate balance between VEGF signaling and MMP activity is essential for adequate fetomaternal angiogenesis, trophoblast invasion, and placental vascular remodeling during organogenesis (Ventureira et al., 2019; Gualdoni et al., 2021).

Conversely, disruption of the VEGF-MMP regulatory axis contributes to abnormal placentation and several forms of vascular placentopathy (Amălinei et al., 2007; Cui et al., 2017; Gualdoni et al., 2021). Altered MMP-2 and MMP-9 expression or activity impairs extracellular matrix remodeling, promotes inflammatory and oxidative pathways, and interferes with spiral artery remodeling, resulting in endothelial dysfunction, increased vascular resistance, placental ischemia, and adverse pregnancy outcomes, including fetal growth restriction, hypertensive disorders of pregnancy, preterm birth, and placental insufficiency (Jacob-Ferreira and Schulz, 2013; Chen et al., 2020). Consistent with these observations, MMP-9-deficient mice exhibit defective ectoplacental cone development, impaired trophoblast differentiation and invasion, reduced implantation success, and smaller litter sizes, underscoring the indispensable role of MMP-9 in normal placental morphogenesis (Plaks et al., 2013).

Nitric oxide (NO) represents one of the principal vasoactive and angiogenic mediators downstream of VEGF signaling during placental development. NO is generated from L-arginine by three nitric oxide synthase (NOS) isoforms: neuronal (nNOS/NOS1), inducible (iNOS/NOS2), and endothelial (eNOS/NOS3) (Hefler et al., 2001; Förstermann and Sessa, 2012; Qian and Fulton, 2013). Whereas nNOS and eNOS are constitutively expressed and their enzymatic activity is calcium-dependent, iNOS is induced under inflammatory or immunological stimuli and produces sustained, high levels of NO independently of intracellular calcium (Förstermann and Sessa, 2012; Eelen et al., 2018). During normal pregnancy, eNOS is the predominant NOS isoform within the placental vasculature and constitutes the major source of physiological NO production. VEGF stimulates eNOS activation through endothelial calcium signaling, while hemodynamic shear stress generated by maternal and fetal blood flow further enhances endothelial NO release (Moncada and Higgs, 2006; Vanhoutte et al., 2016).

Within the uteroplacental circulation, NO is a key regulator of vascular homeostasis. By promoting relaxation of vascular smooth muscle cells, NO maintains low vascular resistance and adequate uteroplacental perfusion, thereby facilitating maternal blood flow to the developing placenta. Beyond its vasodilatory effects, NO contributes to endothelial cell migration, angiogenesis, regulation of vascular permeability, microvascular remodeling, inhibition of platelet aggregation and thrombosis, and maintenance of endothelial function (Krause et al., 2011). During implantation and early placentation, NO also participates in decidualization, extracellular matrix remodeling, and trophoblast invasion by increasing vascular compliance and facilitating spiral artery remodeling, processes that are tightly regulated by oxygen tension, endocrine factors, and oxidative status (Esper et al., 2006; Boeldt et al., 2011; Possomato-Vieira and Khalil, 2016).

The biological importance of NO signaling during placental development is illustrated by studies in eNOS-deficient mice, which exhibit defective spiral artery remodeling, reduced labyrinth development, impaired uteroplacental perfusion, and placental hypoxia, ultimately resulting in fetal growth restriction and abnormal pregnancy outcomes (Kulandavelu et al., 2012; Waker et al., 2023). These findings identify the VEGF-eNOS axis as a central regulatory pathway coupling angiogenesis, vascular remodeling, and placental function throughout gestation.

Reactive oxygen species (ROS) are no longer regarded solely as harmful metabolic by-products but are now recognized as essential signaling molecules that regulate placental angiogenesis, trophoblast differentiation, and vascular homeostasis. During normal placental development, tightly controlled ROS generation modulates multiple intracellular signaling pathways and activates redox-sensitive transcription factors, particularly hypoxia-inducible factors (HIFs), which coordinate gene expression required for trophoblast proliferation, differentiation, and placental vascularization (Bae et al., 2024). In early murine gestation, placental development occurs under a physiological low-oxygen environment resulting from the incomplete remodeling of decidual spiral arteries. This transient hypoxic milieu is indispensable for normal morphogenesis of the ectoplacental cone and subsequent placental development (Patel et al., 2010; Assani et al., 2025; Tan et al., 2025).

Under these conditions, a finely regulated balance between physiological hypoxia, ROS production, and endogenous antioxidant defenses maintains an appropriate redox environment that supports placental growth and angiogenic remodeling (Burton and Jauniaux, 2011; Grzeszczak et al., 2023). Thus, oxygen availability itself represents a major regulator of placental angiogenesis, and precise spatiotemporal modulation of oxygen tension is essential throughout gestation. During early placental development, physiological hypoxia promotes trophoblast proliferation, differentiation, survival, invasion, vasculogenesis, and angiogenesis through activation of HIF-1α, which transcriptionally regulates VEGF and, in part, eNOS, thereby coordinating angiogenic signaling with vascular adaptation. In turn, VEGF-induced eNOS activation increases NO bioavailability, contributing to endothelial function, vasodilation, and vascular remodeling at the maternal-fetal interface. However, these responses are highly dependent on the magnitude and duration of hypoxia. Whereas transient physiological hypoxia is required for normal placental morphogenesis, prolonged or excessive hypoxia induces oxidative stress, impairs trophoblast differentiation, reduces placental and fetal growth, disrupts vascular development, and promotes endothelial dysfunction and apoptosis through activation of stress-related pathways, including p53 and NF-κB. Likewise, although acute hypoxia may transiently enhance eNOS expression and NO production, sustained hypoxia is generally associated with reduced eNOS activity, impaired NO bioavailability, defective placental angiogenesis, and progressive vascular dysfunction.These observations highlight the exquisite oxygen sensitivity of the HIF-VEGF-eNOS signaling axis during placentation and emphasize that disruption of this regulatory network represents a central mechanism underlying placental vascular disease.

The high metabolic activity of the developing placenta, together with increasing maternal oxygen consumption, continuously generates ROS, including superoxide anion (O_2_^-^), as well as NO, which together participate in the regulation of uteroplacental perfusion, endothelial function, and angiogenic signaling (Myatt and Cui, 2004; Assani et al., 2025). At physiological concentrations, ROS interact closely with the VEGF-MMP-eNOS/NO signaling network, contributing to endothelial activation, extracellular matrix remodeling, trophoblast invasion, and vascular adaptation at the maternal-fetal interface. Thus, controlled redox signaling represents an essential component of normal placental angiogenesis rather than merely a consequence of cellular metabolism (Hussain et al., 2021; Li et al., 2025).

However, when ROS production exceeds the antioxidant buffering capacity of placental tissues, oxidative stress develops, disrupting redox-sensitive signaling pathways that are essential for placental development and function. Excessive oxidative stress promotes endothelial dysfunction, inflammation, release of cytokines and anti-angiogenic factors, vasoconstriction, impaired spiral artery remodeling, and increased uteroplacental vascular resistance. Furthermore, mitochondrial dysfunction and altered cellular metabolism establish a self-perpetuating cycle of ROS overproduction, tissue hypoxia, and impaired oxygen utilization, ultimately disturbing HIF-dependent trophoblast gene expression and shifting the balance between trophoblast proliferation and differentiation. These alterations collectively contribute to abnormal placentation, placental vascular insufficiency, fetal growth restriction, and adverse pregnancy outcomes (Li et al., 2025).

Collectively, placental angiogenesis depends on the coordinated integration of the angiogenic system VEGF/VEGFR-MMPs-eNOS/NO-HIFs and physiological redox signaling. Disruption of this complex network, rather than alteration of a single molecular pathway, underlies the development of placental vascular dysfunction and provides a mechanistic framework for understanding how maternal insults, including prenatal alcohol exposure, may induce vascular placentopathy and subsequent embryofetal cardiovascular programming.

## The placenta-embryofetal cardiovascular axis: a developmental framework for cardiovascular programming vulnerability

Under the DOHaD framework, which recognizes the intrauterine environment as a major determinant of adult disease susceptibility (Gluckman and Hanson, 2004; Barker et al., 2010; Eriksson et al., 2011; Burton et al., 2016), the contribution of placental development and function to fetal cardiac morphogenesis has only recently begun to be fully appreciated (Barker et al., 2012). Clinical studies indicate that pregnancies complicated by congenital heart disease (CHD) frequently exhibit placental structural and functional abnormalities, accompanied by altered maternal and fetal circulating angiogenic factors, supporting the concept that placentation and fetal cardiovascular development are closely interconnected biological processes (Llurba et al., 2014; Auger et al., 2015; Ruiz et al., 2016). Likewise, epidemiological human studies have associated placental weight, thickness, and surface area with cardiovascular health later in life (Barker et al., 2012; Eriksson et al., 2011). Complementary evidence from experimental models demonstrates that alterations in vitelline and fetoplacental hemodynamics profoundly influence cardiomyocyte differentiation, cardiac morphogenesis, and fetal heart development (Kowalski et al., 2014; Midgett et al., 2017). Furthermore, pregnancies complicated by fetal growth restriction commonly exhibit defective spiral artery remodeling and impaired labyrinth development (Brosens et al., 2011). These placental vascular abnormalities increase fetoplacental vascular resistance, impair oxygen and nutrient transfer, alter umbilical blood flow, and modify the biomechanical and metabolic environment of the developing fetus, thereby adversely affecting cardiac growth and maturation during critical developmental windows (Burton and Jauniaux, 2011).

Following establishment of the definitive chorioallantoic placenta and maturation of the maternal-fetal circulation (from GD11.5 in the mouse), fetal development enters a second major window of cardiovascular vulnerability (**Figures 1C, d**). During this stage, the placenta becomes fully functional and progressively integrates into a coordinated placenta-embryofetal cardiovascular axis. Concurrently, cardiac development advances through ventricular and atrial septation, valvulogenesis, coronary vascularization, maturation of the conduction system, and functional adaptation of the fetal circulation (**Figure 1**). Unlike the earlier embryonic period (E7-E10.5), these developmental events now occur under conditions in which cardiac completed development and maturation increasingly depends on adequate placental perfusion, oxygen and nutrient availability, endocrine signaling, and fetoplacental hemodynamics. Consequently, disturbances in placental angiogenesis or vascular function during this period may profoundly influence both cardiac morphogenesis and long-term cardiovascular programming.

One of the strongest experimental demonstrations of this functional dependence is provided by the regulation of ventricular myocardial growth through placental-derived metabolic signals. Among the different placental-derived signals proposed to regulate fetal cardiogenesis, the IGF2 pathway currently provides one of the best-characterized mechanistic examples linking placental function with myocardial growth. In mice, between GD10 and GD14.5, epicardial expression of insulin-like growth factor-2 (IGF2), a major mitogenic factor controlling ventricular cardiomyocyte proliferation, is regulated by placental oxygen and glucose availability (Shen et al., 2015). During early fetal development, IGF2 expression is initially stimulated through erythropoietin-dependent signaling; however, following maturation of the placental circulation, oxygen and glucose become its principal physiological regulators. Accordingly, experimental models exhibiting impaired placental vascular development and fetal hypoglycemia show reduced epicardial IGF2 expression, ventricular myocardial hypoplasia, and peripheral edema despite the absence of primary cardiac malformations (Shen et al., 2015). Ex vivo studies further demonstrate that oxygen tension and glucose availability directly regulate epicardial IGF2 expression (Shen et al., 2015). Collectively, these findings provide compelling evidence that normal myocardial growth during mid-gestation depends critically on placental function rather than exclusively on intrinsic cardiac developmental programs. This experimental paradigm represents one of the clearest biological demonstrations that the placenta actively regulates fetal cardiac morphogenesis, supporting the concept that the placenta functions as a developmental signaling organ rather than merely a passive interface for maternal-fetal exchange.

Collectively, the available experimental, clinical, and epidemiological evidence supports the concept of the placenta-embryofetal cardiovascular axis, whereby placental vascular development and fetal cardiogenesis constitute a single integrated developmental system rather than independent biological processes (Linask, 2013, 2014). Within this framework, the placenta coordinates the angiogenic, metabolic, endocrine, oxygen-sensitive, and hemodynamic signals that govern fetal cardiovascular maturation. Accordingly, placental vascular dysfunction should be considered a primary mechanistic intermediary linking adverse intrauterine exposures with altered cardiac developmental programming. This integrated model also provides the biological rationale for understanding how maternal alcohol exposure may impair cardiovascular development not only through the direct embryotoxic actions of ethanol but also indirectly through alcohol-induced placental vascular dysfunction, thereby increasing susceptibility to cardiovascular disease throughout postnatal life.

## Fetal cardiovasculopathy programming by maternal alcohol intake

The heart is the first functional organ to develop during embryogenesis and one of the earliest targets of prenatal alcohol exposure because the initial stages of cardiogenesis coincide with critical developmental windows of ethanol susceptibility. Consequently, disturbances occurring during these early periods may have profound effects on both cardiac morphogenesis and long-term cardiovascular function.

Clinical evidence indicates that congenital heart defects (CHDs) are ones of the major non-neurological manifestations of fetal alcohol spectrum disorders (FASD), occurring in approximately 30% of affected children and in up to 50% of those diagnosed with fetal alcohol syndrome (FAS) (Abel, 1990; Burd et al., 2007). Reported abnormalities include atrial and ventricular septal defects, valvular malformations, pulmonary artery stenosis, Tetralogy of Fallot, transposition of the great arteries, and other structural cardiac anomalies (Grewal et al., 2008). In addition, epidemiological evidence suggests that prenatal alcohol exposure may also predispose individuals to cardiovascular dysfunction later in life, including hypertension, vascular dysfunction, increased arterial stiffness, cardiometabolic abnormalities, and an elevated risk of adult cardiovascular disease (Grewal et al., 2008). However, more subtle alterations, such as impaired ventricular growth, myocardial remodeling, or reduced cardiac compliance, may remain underrecognized because they are less frequently evaluated in clinical studies.

Despite the broad spectrum of cardiovascular abnormalities associated with prenatal alcohol exposure and FASD and even the etiology of alcohol-induced CHDs has been the focus of several studies, relatively few have specifically investigated the developmental origins, critical windows of vulnerability, and molecular mechanisms underlying alcohol-induced embryofetal cardiac injury (Cavieres and Smith, 2000). Consequently, much of our current understanding derives from indirect evidence or from studies primarily focused on other components of FASD, highlighting an important gap in the field. Some studies examining the risks between maternal alcohol consumption and most CHDs showed findings with many limitations regarding retrospective exposure assessment and suggested controverty results because of no statistically significances or unmeasured confounded variables (Zhu et al., 2015). Therefore, it was concluded that additional studies with improvement in measurement of alcohol consumption were recommended.

The direction of prenatal alcohol-cardiogenesis changes appears to be depend on the developmental window, experimental model, and exposure paradigm. Experimental prenatal alcohol studies were generally focused on descriptive analyses of exposure effects beginning on early mouse embryonic days (around gastrulation). Reviewing the background on the prenatal ethanol exposure on myocardial growth in experimental murine models, data relating to timing of exposures and induced mechanisms are very limited. For example, Peng et al. (2015) reported ventricular hypertrophy in E17.5 fetuses from C57Bl/6 mice exposed to 56% (v/v) ethanol (5 ml/kg/day by gavage) between E8.5 and E16.5. This phenotype was associated with increased histone H3 acetylation at promoters of genes involved in cardiac development, suggesting that epigenetic dysregulation contributes to alcohol-induced myocardial remodeling. Conversely, other experimental observations support the hypothesis that prenatal alcohol exposure may impair ventricular growth, leading to myocardial hypoplasia rather than hypertrophy. These available evidence suggests that alcohol does not induce a single cardiac phenotype but rather modulates myocardial growth according to the specific developmental context in which exposure occurs.These apparently divergent findings probably reflect differences in all variables of ethanol exposure, including the timing, duration, dose, and pattern of ethanol exposure, as well as differences among experimental models and developmental endpoints evaluated. Evidence obtained from complementary experimental models further supports this concept. Using human induced pluripotent stem cell-derived cardiomyocytes (hiPSC-CMs), ethanol exposure (17–100 mM) impaired early cardiac differentiation and reduced cardiomyocyte proliferation in a dose-dependent manner, resulting in fewer cardiac progenitor cells and mature cardiomyocytes (Man et al., 2025).

Consistent with these observations, our murine model of perigestational moderate alcohol exposure from previous gestation, demonstrated that ethanol administration until GD10 disrupts embryonic organogenesis by altering differentiation, growth and morphology, including that of the neural tube development (Coll et al., 2011), through dysregulation of prostaglandin metabolism signaling pathways (Cebral et al., 2007). Moreover, perigestational alcohol intake up to organogenesis altered the expression and tissue distribution and expression of cadherins within the ectoderm and somitic and cardiac mesoderm (Coll et al., 2011), leading to impaired endocardial integrity and reduced ventricular myocardial compaction. Because cadherins play fundamental roles in cell recognition, epithelial organization, tissue adhesion, and epithelial-to-mesenchymal transition during gastrulation and organogenesis, disruption of their expression in early embryos provides a plausible mechanism linking maternal ethanol exposure with abnormal myocardial architecture from early stages of organogenic development. In addition, these adherent protein alterations were accompanied by impaired embryionic cellular proliferation and increased apoptosis (Coll et al., 2017), suggesting that these altered pathways, impacting to cell and tissue integrity, may compromise early events of myocardic development during stages of cardiac tube fusion (mouse E9), looping (E9.5) and morphogenesis (E10) and remodeling (E10.5) of organogenic heart.

Comparable developmental abnormalities have also been reported in zebrafish embryos exposed to ethanol between 3 and 24 hours post-fertilization. In this model, ethanol reduced cardiac chamber size, decreased the number of myocardial cells, induced myocardial fibrosis, and delayed the differentiation of cardiomyocytes derived from both the first and second heart fields (Sarmah et al., 2017). Although zebrafish and mammalian cardiogenesis differ in several developmental aspects, these findings support the concept that multiple cardiogenic progenitor populations are highly sensitive to ethanol during early cardiac specification.

Collectively, the available experimental evidence suggests that prenatal alcohol exposure interferes with multiple regulatory processes controlling myocardial development, including epigenetic regulation, cellular metabolism, oxidative homeostasis, cell adhesion, proliferation, differentiation, and apoptosis. However, the resulting cardiac phenotype appears to be highly context-dependent, and the apparent discrepancies among studies most likely reflect differences in developmental timing, exposure paradigms, species, and experimental approaches. Consequently, whether prenatal alcohol exposure ultimately leads to myocardial hypoplasia, hypertrophy, or mixed remodeling phenotypes remains incompletely resolved, highlighting the need for studies that directly compare exposure windows and integrate placental, molecular, and functional mechanisms within the same experimental framework.

Beyond its effects on ventricular wall growth, experimental evidence suggests that prenatal alcohol exposure may also interfere with ventricular trabeculation, another critical morphogenetic process occurring during early fetal cardiogenesis (approximately from mouse GD10 onward). Trabecular formation depends on the coordinated proliferation, migration, and differentiation of cardiomyocytes, as well as on tightly regulated interactions between the endocardium and myocardium. Consequently, disruption of any of these processes during critical developmental windows may compromise ventricular maturation and subsequent cardiac function. Experimental studies support this possibility. In mice, acute ethanol exposure induced by intraperitoneal administration at GD6.75 resulted in reduced ventricular trabeculation in E15.5 fetuses (Serrano et al., 2010), suggesting that early alcohol exposure can exert persistent effects on later stages of cardiac morphogenesis. Although the mechanisms responsible for this phenotype remain incompletely understood, reduced myocardial trabeculation and growth may be associated to impaired cardiomyocyte proliferation at early stages of development.

Another potential celular mechanism associated to alcohol-exposed effects involves altered cell migration. Proper ventricular trabeculation requires coordinated migration and rearrangement of myocardial cells during chamber morphogenesis. Although direct evidence linking ethanol-induced migratory defects with impaired trabeculation remains limited, numerous experimental studies have demonstrated that ethanol disrupts cellular migration in different biological systems. For example, chick embryos exposed to ethanol immediately after neural crest cell migration exhibited delayed and abnormal migration toward the trunk, an effect that persisted for at least 24 hours after ethanol removal (Rovasio and Battiato, 2002).

Collectively, these observations support the hypothesis that ethanol may impair ventricular trabeculation by disrupting fundamental cellular behaviors required for cardiac morphogenesis, including cardiomyocyte proliferation and migration. However, current evidence remains largely indirect, and whether these mechanisms operate specifically within the developing myocardium has not yet been conclusively demonstrated. Future studies integrating cellular dynamics, myocardial morphogenesis, and their relationship with placental physiology will be necessary to establish the relative contribution of these mechanisms to alcohol-induced cardiac malformations.

One aspect that has not been thoroughly investigated due to technological limitations is the cardiac function and its role in abnormal heart development in the progression to explain the FAS-related CHDs. However, the nature and timing of these functional alterations vary considerably among experimental models, probably reflecting differences in species, ethanol dose, exposure paradigm, developmental window, and the age at which cardiovascular function is evaluated.

Beyond structural abnormalities, some experimental evidence indicates that prenatal alcohol exposure at gastrulation impairs the cardiac function, for example by abnormal valvulogenesis, at late stages. In C57BL/6J mice, administration of two intraperitoneal ethanol injections (2.9 g/kg) at GD6.75 produced fetuses examined at E15.5 that exhibited semilunar valve abnormalities together with significant functional cardiac impairment, detected by Doppler echocardiography, including increased heart rate, prolonged isovolumic contraction and relaxation times, and an elevated myocardial performance index, indicating global myocardial dysfunction (Serrano et al., 2010). Using the same exposure paradigm, neonatal mice, examined five days after birth, displayed ventricular wall thinning, a reduced collagen I/III ratio consistent with myocardial fibrosis, increased left ventricular wall stress during diastole, impaired diastolic function, and decreased ejection fraction and fractional shortening, demonstrating that a transient embryonic exposure can produce persistent structural and functional cardiac abnormalities extending into postnatal life (Ninh et al., 2019).

Conversely, studies using prolonged maternal alcohol exposure throughout gestation have reported different outcomes. In Sprague-Dawley rats exposed to a 6% (v/v) ethanol liquid diet during pregnancy, no significant cardiac abnormalities were detected either at term or in 30-day-old offspring. However, adult offspring examined at eight months developed left ventricular hypertrophy, increased myocardial fibrosis, and reduced aortic flow velocity, suggesting progressive ventricular dysfunction and/or vascular impairment that became evident only later in life (Nguyen et al., 2014). These apparently discrepant findings may reflect differences in exposure timing, cumulative dose, species-specific susceptibility, and developmental adaptation. Whereas acute exposure during critical windows of cardiac morphogenesis primarily affects organogenesis, prolonged lower-level exposure may induce more subtle developmental changes that become clinically apparent only during postnatal aging.

Experimental studies have also demonstrated that prenatal alcohol exposure alters fetal hemodynamics independently of overt structural cardiac defects. In chick embryos, ethanol exposure increased retrograde blood flow and impaired valvular function (Karunamuni et al., 2014). Likewise, in a murine model designed to mimic second-trimester exposure, administration of ethanol at GD12.5 produced acute reductions in fetal cardiac output acceleration together with altered blood flow in the umbilical artery, carotid artery, middle cerebral artery, and peripheral circulation, as well as decreased velocity-time integral in most vascular beds and abnormal retrograde cerebral blood flow (Bake et al., 2012). Notably, these hemodynamic disturbances persisted for several days after ethanol exposure, suggesting that even transient prenatal exposure may compromise fetal perfusion during critical periods of organ maturation.

Collectively, these experimental observations support the hypothesis that prenatal alcohol exposure disrupts not only cardiac morphogenesis but also the biomechanical environment in which the fetal heart develops. Because myocardial growth, valve remodeling, and vascular maturation are highly dependent on blood flow-derived mechanical forces, alcohol-induced alterations in fetal hemodynamics may secondarily interfere with mechanotransduction pathways regulating cardiovascular development (Duchemin et al., 2019; Jarrell et al., 2019). Nevertheless, the relative contribution of primary myocardial injury versus secondary hemodynamic disturbances remains incompletely understood. Future studies integrating cardiac imaging, placental function, and fetal circulation will be required to determine how these mechanisms interact throughout gestation.

An important limitation shared by most available experimental studies is that cardiac function has generally been evaluated without simultaneous assessment of placental vascular function or fetoplacental hemodynamics. Consequently, although these studies clearly demonstrate alcohol-induced fetal cardiovascular dysfunction, the extent to which placental vascular impairment contributes to these abnormalities remains largely unresolved. Addressing this gap represents one of the principal challenges for future translational studies.

Current mechanistic knowledge of alcohol-induced congenital heart defects remains surprisingly limited. There are several hypotheses for how alcohol disrupts development, and many mechanisms have been implicated, including effects on lipid metabolism, zinc, lipid rafts, cell adhesion molecule, alcohol dehydrogenase, catalase, and lipid related gene expression and genes associated with the folic acid cycle in abnormally developing hearts and placentas (Lindi et al., 2001; Goodlett et al., 2005; Serrano et al., 2010; Han et al., 2016). Ethanol also induced mitochondrial dysfunction, reduced membrane potential, decreased mitochondrial content, impaired cellular respiration, and dysregulation of proteins involved in fatty acid oxidation, indicating that metabolic impairment represents an additional mechanism through which ethanol compromises cardiac development (Grewal et al., 2008).

However, maternal alcohol consumption is widely recognized as a major inducer of oxidative stress during embryonic and fetal development through disruption of cellular redox homeostasis during ethanol metabolism. Excessive generation of reactive oxygen and nitrogen species (ROS/RNS), together with acetaldehyde accumulation and alterations in the NADH/NAD^+^ redox balance, promotes oxidative damage to DNA, proteins, membrane lipids, and mitochondria, thereby disrupting multiple signaling pathways required for normal embryonic development (Kotch et al., 1995; Wells et al., 2009). Extensive experimental evidence has demonstrated alcohol-induced oxidative injury in whole embryos and numerous developing organs (Kotch et al., 1995; Chu et al., 2007; Dong et al., 2010; Coll et al., 2017), establishing oxidative stress as one of the principal pathogenic mechanisms underlying fetal alcohol spectrum disorders (FASD). However, compared with the central nervous system, considerably less attention has been devoted to understanding how oxidative stress contributes to embryofetal cardiac development. Experimental studies support this concept. In Wistar rats exposed to ethanol from gestational day 7 until postnatal day 21, offspring exhibited sustained cardiac oxidative damage and inflammation at both juvenile (21 days) and adult (90 days) ages, whereas vitamin E supplementation markedly attenuated these abnormalities, supporting a causal role for oxidative stress in alcohol-induced cardiac injury (Shirpoor et al., 2015). Likewise, in our murine model of perigestational alcohol consumption, organogenic embryos (E10) displayed reduced catalase activity, increased lipid peroxidation, and enhanced protein nitration associated with elevated nitric oxide levels, demonstrating an early oxidative imbalance that was closely associated with embryonic dysmorphogenesis (Coll et al., 2017). Additional experimental studies have further linked prenatal alcohol-induced oxidative stress with dysregulated prostaglandin signaling (Cebral et al., 2007), disturbances in cAMP-dependent pathways and protein metabolism (Dong et al., 2010), highlighting the broad molecular consequences of ethanol-induced redox imbalance during organogenesis.

Oxidative stress is also closely associated with activation of apoptotic pathways, one of the most consistently reported cellular responses to prenatal alcohol exposure across experimental models (Zhang et al., 2017). Experimental evidence indicates that excessive ROS/RNS production disrupts redox-sensitive signaling pathways controlling cell survival, thereby promoting cardiomyocyte apoptosis during critical stages of heart development. In a murine model in which pregnant females received ethanol by gavage between E8.5 and E16.5, fetal hearts analyzed at E17.5 exhibited increased expression of caspase-3 and caspase-8, a higher number of TUNEL-positive cardiomyocytes, and reduced expression of the anti-apoptotic protein Bcl-2 (Yan et al., 2017). Likewise, in our murine model of perigestational alcohol consumption until GD10, a significant increase in TUNEL-positive cells was observed within the embryonic myocardium during organogenesis (Coll et al., 2017). Although these findings consistently support apoptosis as an important mechanism of alcohol-induced cardiac injury, the upstream molecular pathways linking oxidative stress with cardiomyocyte loss remain incompletely understood.

Beyond apoptosis, accumulating experimental evidence suggests that disruption of other cellular homeostatic mechanisms, particularly autophagy, may also contribute to alcohol-induced cardiac developmental defects. Physiological autophagy plays essential roles during early cardiogenesis by regulating cardiomyocyte differentiation, migration, and heart tube formation (Lu et al., 2014; Wang et al., 2015). In chick embryos, exposure to 2% ethanol increased autophagic activity in cardiac progenitor cells, impairing their migratory and differentiation capacities and resulting in cardia bifida (Li et al., 2015). However, evidence supporting a direct contribution of dysregulated autophagy to mammalian fetal cardiac abnormalities remains limited, and the interaction between oxidative stress, apoptosis, and autophagic pathways during prenatal alcohol exposure has not yet been fully elucidated.

Collectively, the available evidence suggests that oxidative stress generated during ethanol metabolism represents a major upstream mechanism capable of disrupting the integrated angiogenic signaling network that regulates embryofetal cardiovascular development. Because normal cardiogenesis depends on the coordinated activity of redox-sensitive pathways, including VEGF, NO, HIF-1, and related angiogenic mediators, disturbances in physiological redox homeostasis may interfere with endothelial differentiation, myocardial growth, vascular remodeling, and cardiac maturation. Although the precise molecular interactions remain incompletely understood, these observations provide a plausible mechanistic link between prenatal alcohol-induced oxidative stress, dysregulation of angiogenic signaling, and long-term cardiovascular programming.

Through activation of VEGFR2/KDR, the differentiation and renewal of cardiomyocytes in the developing heart are critically regulated by the VEGF signaling pathway (Chen et al., 2006). Accordingly, precise temporal and spatial regulation of VEGF expression is essential for normal myocardial growth, ventricular wall compaction, endocardial cushion formation, and cardiac morphogenesis. Experimental evidence indicates that prenatal alcohol exposure interferes with these VEGF-dependent developmental processes. In chick embryos exposed to 2% ethanol, severe cardiac abnormalities, including cardia bifida, were associated with impaired differentiation and migration of cardiac progenitor cells and reduced expression of key cardiogenic genes, including BMP-2, FGF8, and VEGF (Li et al., 2015). The biological relevance of this angiogenic pathway is further supported by genetic models. Myocardial VEGF-null mice develop progressive ventricular and atrial hypoplasia by embryonic day E13.5 despite apparently normal cardiac activity at E12.5, demonstrating that adequate VEGF signaling is indispensable for myocardial growth during fetal development (Giordano et al., 2001). Importantly, both insufficient and excessive VEGF activity appear to be detrimental for cardiac morphogenesis, indicating that cardiogenesis depends on tightly regulated VEGF signaling gradients rather than simply high VEGF expression. Likewise, experimental hypoxia may induce VEGF overexpression within the endocardial cushions, inhibiting the endothelial-to-mesenchymal transition and producing malformed cardiac valves (Dor et al., 2003). Collectively, these findings suggest that prenatal alcohol exposure may disrupt the finely regulated VEGF signaling environment required for normal cardiac morphogenesis, thereby affecting myocardial growth, endocardial cushion remodeling, and valvulogenesis during critical windows of embryonic development.

Ethanol metabolism may interfere with VEGFR2 signaling during critical windows of development through redox-dependent mechanisms, affecting ventricular growth and cardiac morphogenesis. In cultured brain microvascular endothelial cells, ethanol induced ligand-independent activation of receptor tyrosine kinases, including VEGFR2/KDR, an effect that was prevented by inhibitors of ethanol metabolism and antioxidant treatment (Haorah et al., 2008). These observations suggest that ethanol-derived oxidative stress may induce abnormal VEGFR2 phosphorylation and disrupt the physiological coordination between VEGF availability and receptor activation. Because physiological VEGF signaling itself requires controlled ROS generation through NADPH oxidase to promote VEGFR2 autophosphorylation (Ushio-Fukai, 2006), excessive oxidative stress generated during ethanol metabolism may convert this tightly regulated redox signaling into pathological activation in the developing heart, thereby impairing downstream angiogenic responses.

Alterations of the VEGF/VEGFR2 signaling axis are expected to affect multiple downstream cadiac effectors, particularly MMPs and NOS) Consistent with this concept, ethanol-induced VEGFR2 activation in endothelial cells was accompanied by increased expression and activation of MMP-2 and MMP-9, together with phosphorylation of the tight-junction proteins occludin, ZO-1, and claudin-5, ultimately increasing endothelial permeability (Haorah et al., 2008). However, the effects of maternal alcohol intake on the expression and activity of MMPs in the embryofetal heart are still poorly understood. In other experiments of ischemia-reperfusion, oxidative and nitrosative stress promotes intracellular activation of pro-MMP-2 through peroxynitrite generation, resulting in proteolytic degradation of sarcomeric and mitochondrial proteins (Cheung et al., 2000; Wang et al., 2002). Intracellular active MMP-2 is normally associated with myofibrils and mitochondria, where it contributes to myocardial homeostasis (DeCoux et al., 2014). Therefore, maternal alcohol exposure, via induction of oxidative stress, may aberrantly activate intracellular MMP-2 during fetal development, promoting mitochondrial damage and dysfunction, sarcomeric disorganization and apoptosis thereby contributing to structural and functional abnormalities of the developing myocardium (Lovett et al., 2012).

Collectively, the available experimental evidence indicates that prenatal alcohol exposure disrupts multiple interconnected processes governing cardiac morphogenesis, including cardiomyocyte proliferation and differentiation, redox homeostasis, apoptosis, angiogenic signaling, extracellular matrix remodeling, and fetal hemodynamics. However, these direct effects on the developing heart alone are unlikely to fully explain the diversity, severity, and long-term persistence of alcohol-induced cardiovascular abnormalities. Because many of the molecular pathways affected by maternal ethanol exposure are also fundamental regulators of placental angiogenesis and vascular development, increasing evidence supports the concept that placental dysfunction represents a critical mechanistic intermediary linking maternal alcohol exposure with fetal cardiac injury. Consequently, understanding how prenatal alcohol exposure alters placental vascular development and function has become essential for elucidating important mechanisms underlying embryofetal cardiovascular programming. Accordingly, the next section focuses on placental vascular development as a central biological target of prenatal alcohol exposure and discusses how alcohol-induced placental vascular dysfunction may contribute to fetal cardiovascular programming.

## Placental vascular disruptions by maternal alcohol exposure

Placental development and vascular integrity are essential for maintaining fetal homeostasis and supporting normal cardiovascular development. Consequently, disturbances in placental morphogenesis or function during critical windows of gestation may permanently modify the intrauterine environment and increase susceptibility to cardiovascular disease later in life. Maternal alcohol consumption is now recognized as an important cause of placental dysfunction, associated with disrupted morphogenesis, endocrine activity, metabolic homeostasis, and abnormal transport of oxygen, nutrients, iron, folate, and metabolic waste products (Gundogan et al., 2008, 2015; Ramadoss and Magness, 2012a; Hutson et al., 2012; Gupta et al., 2016; Kwan et al., 2020). These placental alterations placenta contribute to fetal growth restriction, perinatal morbidity and mortality, several of the clinical manifestations that characterize fetal alcohol spectrum disorders, and predispose to heart disease programming and persistent cardiovasculopathy susceptibility in postnatal life (Aliyu et al., 2008, 2011; Salihu et al., 2011; Lacko et al., 2017).

Gestational alcohol intake has been associated with the so-called *alcohol-related placental-associated syndrome*, encompassing miscarriage, placental abruption, placental insufficiency, preeclampsia, placenta previa, placenta accreta, placental ischemia and infarction, preterm birth, gestational hypertension, and fetal growth restriction (Burd et al., 2007; Gundogan et al., 2010; Meyer-Leu et al., 2011; Salihu et al., 2011; Avalos et al., 2014; Carter et al., 2016; Tai et al., 2017; Orzabal et al., 2019; Ohira et al., 2019; Odendaal et al., 2020). Clinical and experimental studies indicate that the placental vasculature represents one of the primary targets of prenatal alcohol exposure. In both humans and experimental animal models, alcohol exposure frequently impairs maternal angiogenesis, uteroplacental perfusion, vascular remodeling, and placental hemodynamics, resulting in increased vascular resistance, malperfusion, placental vasculopathy, and impaired umbilical circulation (Iveli et al., 2007; Gundogan et al., 2008, 2015; Ramadoss and Magness, 2012a; Khong et al., 2016; Gyselaers, 2023). Prenatal alcohol exposure alters the placental iron transport yielding to fetal iron deficiency anemia, condition that exacerbates alcohol-related growth restriction (Kwan et al., 2020). Chronic and heavy alcohol use in pregnancy may impair placental transport of folic acid y decreasing expression of transport proteins, thus contributing to the deficits observed in FASD (Hutson et al., 2012). Therefore the most generalized effects of gestational alcohol intake on the placenta by which maternal-fetal circulatory and uteroplacental vascular hemodynamics may be affected during gestation, were reported to be on its vascularization.

Emerging experimental evidence suggests that the pathogenic origin of placental abnormalities lies in early gestation, during peri-implantation, gastrulation, and organogenesis, when trophoblast differentiation, decidual remodeling, and placental angiogenesis are particularly sensitive to alcohol exposure (Livy et al., 2004). In agreement with this concept, recent mouse studies have demonstrated that perigestational alcohol consumption disrupts early decidual angiogenesis, trophoblast development, and remodeling of the maternal-fetal interface, supporting the existence of critical windows of placental susceptibility to alcohol (Coll et al., 2018; Ventureira et al., 2019; Gualdoni et al., 2021). Accordingly, following it is reviewed the current evidence on the effects of maternal alcohol consumption on placental angiogenesis from two perspectives: (i) alterations affecting the maternal decidual vasculature and uteroplacental remodeling, and (ii) abnormalities involving trophoblast differentiation/invasion, fetal placental vascularization, and remodeling of the maternal-fetal interface (**Table 1**). Together, these mechanisms provide the biological framework linking placental dysfunction with fetal cardiovascular programming induced by prenatal alcohol exposure.

**Table 1:** Summary of the main findings of maternal alcohol exposure on placenta and materno-fetal vascularization-angiogenesis.

Alcohol exposure pattern	Ethanol exposure values	Time of exposure	Placental vascularization effects	References
Rat Model				
Chronic alcohol gestational exposure	8-18-24-37% EDC	Gestational days 6-16	Placental oxidative stress and mitochondrial dysfunction Incomplete matemal decidual artery remodeling Decreased uterine artery function and blood flow Placental vasoconstriction-Hypoxia Altered labyrinthine layer Disrupted spiral artery vascular muscular layer Impaired physiological conversion of maternal uterine vasculature	(Gundogan et al., 2008, 2010, 2015; Ramadoss and Magness 2012a)
Chronic alcohol perigestational exposure	10 to 30% v/v ethanol	1 month before and during gestation	Decreased placental blood flow	(Ramadoss and Magness 2012a)
Chronic alcohol gestational exposure	4.5g/kg dose (BAC: 216 mg/dl) (dally orogastric gavage)	Gestational day 5 to 19.	Abnormal uterine arteriolar NO production and pathway Impaired uterine vascular remodeling Reduced uterine artery eNOS expression and activity Decreased uterine vasodilation. Upregulated MMP-2 and MMP-9 Diminished uterine artery function and altered proteome	(Orzabal et al., 2019)
Chronic alcohol gestational exposure (chronic binge-like alcohol)	4.5 g ethanol/kg/day (BAC: 216 mg/dl)	Gestational days 7 to 17	Impaired maternal uterine artery reactivity Decreased uterine vasodilation Uterine vascular dysfunction Altered uterine artery vasodilation	(Subramanian et al., 2014; Ramadoss and Magness 2012a)
Chronic alcohol gestational exposure	18-24-37% EDC	Gestational days 6-16	Incompleted uterine vascular transformation Abnormal placental morphogenesis Abnormal placental metabolism and hormone function	(Gundogan et al., 2008; 2010; 2015)
Mouse Model				
Chronic alcohol gestational exposure	(BAC 110 mg/dL)	Gestational days 6-18	Placental resistance Abnomal vascular perfusion Reduced NO modulation of the mesenteric artery	(Ramadoss and Magness 2012a)
Perigestational alcohol exposure up to gestalonal days 4-5	10% ethanol/drinking water (BAC 22-24 mg/dl)	15 days before +4-5 days of gestation	Desregulation of Implantation timing (*In vitro*) Altered GD5-trophoblast development and differentiation Altered GD5-trophoblastic function (outgrowth)	(Pérez-Tito et al., 2014)
Perigestational alcohol exposure up to gestational day 10	10% ethanol/drinking water (BAC 22-24 mg/dl)	15 days before +10 days of gestation	Altered decidual vascularization-angiogenesis Reduced maternal VEGF/KDR expression Increased decidual antiangiogenic factors (FLT-1) Reduced decidual endothelial proliferation Increased oxidative stress in matemo-decidual tissue Reduced endothelial NOS expression Reduced labyrinth Abnormal juntional trophoblastic cells Increased hypoxia at decidual-trophoblastic tissues Altered trophoblastic VEGF/R/NOS/MMPs expression	(Ventureira et al., 2019; Coll et al., 2018; Gualdoni et al., 2021)
Human				
Chronic alcohol gestational exposure	20 g ethanol/day (10 standard drinks/week).	During early gestation	Diminished placental vascular density Decreased KDR expression in placenta at term	(Holbrook et al., 2019)
Semi-chronic alcohol gestational exposure	<1.5 or +1.5 drinks/week (drinking frequency: <3 or 3+ days/week)	Until the second/third trimester	Placental abnormality- Altered NO	(Ohira et al., 2019)
Chronic alcohol gestational exposure			Altered maternal uterine artery transcriptome Altered transcription of proteases/matrix proteins Altered uterine vascular remodeling Altered uterine angiogenic mRNA transcriptome	(Ramadoss and Magness 2012a)
Chronic alcohol gestational exposure	Moderate-high concentrations	Along gestation	Impaired blood flow/artery vasodilatation Abnormal nutrient transport Umbilical cord vasoconstriction	(Burd & Hofer 2008)
Chronic alcohol gestational exposure	Average, 200-250 mL ethanol/trim	Along gestation	Altered contractility of umbilical cord artery. Altered vasculature, uteroplacental malperfusion, resistance Inhibition of NO release and prostacydin	(Iveli et al., 2007)
Chronic-semichronic alcohol gestational	Heavy, moderate and/or light drinking	2nd, 3rd, 1st+3rd or 2nd+3rd trimesters	Utero-placental malperfusion and hypoplasia Premature delivery	(Tai et al., 2017)
Acute alcohol gestational exposure	8 drinks (One binge-heavy)	1.5 days/week during gestation	Decreased placental growth	(Carter et al., 2016)
Chronic alcohol gestational exposure	Two drinks (wine)/day (18-30 g ethanol/day) (BAC 5-100 mg/dL)	Along gestation	Placental abruption	(Burd et al., 2007)
Chronic alcohol gestational exposure	Moderate quantities	Along gestation	Reduced placental weight Impaired blood flow/artery vasodilatation Abnormal nutrient transport Umbilical cord vasoconstriction	(Burd & Hofer 2008; Ramadoss and Magness 2012a, 2012b; Gundogan et al., 2015)
Chronic alcohol gestational exposure	High alcohol quantities	Along gestation	Reduced blood flow and nutrient interchange	(Gundogan et al., 2008, 2010, 2015)
Chronic alcohol gestational exposure	Two drinks (wine)/day (BAC 5-100 mg/dL) (18-30 g ethanol/day)	Along gestation	Placental abruption	(Burd et al., 2007)
Chronic alcohol gestational exposure	Heavy, moderate and/or light drinking	Along gestation, or during: 2nd, 3rd, 1st+3rd or 2nd+3rd trimesters	Utero-placental malperfusion and hypoplasia	(Tai et al., 2017)
Chronic alcohol gestational exposure	High quantities-severe Intake	Along gestation	Vascular resistance, vasoconstriction	(Ramadoss et al., 2011; Bosco and Diaz, 2012)
Chronic alcohol gestational exposure		Chronic binge-like during gestation	Impaired maternal uterine artery reactivity Vascular dysfunction Decreased uterine vasodilation	(Subramanian et al., 2014)

Maternal decidual vasculature and trophoblast remodeling are primary targets of gestational alcohol exposure. Experimental and clinical evidence indicates that the maternal decidual vasculature is one of the earliest placental targets of gestational alcohol exposure. Chronic gestational ethanol intake could disrupt maternal decidual spiral artery remodeling by which the interaction between the invasive trophoblasts and maternal vessels is impaired, thus leading to altered placental blood flow and nutrient exchange. During third trimester of gestation, alcohol affects the uteroplacental vascular function (Rosenberg et al., 2010; Subramanian et al., 2014; Orzabal et al., 2019), uterine spiral artery remodeling, angiogenesis and vasodilation (Radek et al., 2005), via altered endothelial angiogenic gene expression (Ramadoss and Magness, 2012b) and endothelial proteome defects (Ramadoss et al., 2011; Ramadoss and Magness, 2012a), ultimately increasing uteroplacental vascular resistance, reducing placental perfusion, and impaired blood flow and nutrient exchange across the placenta (Radek et al., 2005; Gundogan et al., 2008, 2010; Rosenberg et al., 2010; Ramadoss and Magness, 2011; Ramadoss and Magness, 2012a, 2012b, 2012c, Subramanian et al., 2014; Orzabal et al., 2019). Consistent with these observations, we recently demonstrated that perigestational alcohol consumption disrupts decidual angiogenesis during the trophoblast-independent phase of placentation, suggesting that deficient maternal vascular development may represent one of the earliest pathogenic events preceding trophoblast invasión and maternal vascular remodeling (Ventureira et al., 2019).

Accumulating evidence further indicates that uteroplacental vascular dysfunction is closely linked to impaired trophoblast development and function. Several studies have proposed that defective trophoblast differentiation, migration, invasion, and endothelial replacement of maternal spiral arteries constitute major mechanisms underlying alcohol-induced placental vasculopathy (Han et al., 2012; Gundogan et al., 2008, 2010; Radek et al., 2005; Rosenberg et al., 2010; Ramadoss and Magness, 2012b, 2012c; Subramanian et al., 2014; Orzabal et al., 2019). In animal models, chronic ethanol exposure consistently impairs maternal spiral artery remodeling by reducing trophoblast invasion, thereby compromising placental blood flow and maternal-fetal nutrient exchange (Gundogan et al., 2008, 2010). Also, moderate and high ethanol exposure could reduce labyrinth growth and branching morphogenesis, even low ethanol doses induce disorganization of labyrinthine vascular channels, indicating that placental angiogenesis is highly sensitive to alcohol and that these effects can occur in a dose-dependent manner. Within the junctional zone, alcohol may decrease trophoblast giant cell and glycogen cell populations and impairs maternal vascular transformation at the trophoblast-decidual interface (Gundogan et al., 2008, 2010, 2013, 2015). The reduction in invasive trophoblast populations parallels the severity of maternal vascular remodeling defects, suggesting that abnormal trophoblast differentiation and placental vascularization may be mechanistically coupled.

More recent studies suggest that these pathogenic events may originate much earlier than previously recognized. Perigestational alcohol consumption, restricted to the peri-implantation period or extending only until early organogenesis, is sufficient to induce persistent defects in trophoblast development, labyrinthine vascularization, and placental branching morphogenesis later in gestation (Gårdebjer et al., 2014; Kalisch-Smith and Moritz, 2017; Kalisch-Smith et al., 2019; Gualdoni et al., 2021, 2022a). Likewise, our studies demonstrated that alcohol exposure during peri-implantation disrupts early trophoblast differentiation and subsequent labyrinth vascular development, supporting the concept that alcohol-induced placental insufficiency and uteroplacental vasculopathy originate during the earliest stages of placentation, well before the establishment of the definitive maternal-fetal circulation (Burton and Jauniaux, 2010; Pérez-Tito et al., 2014; Ventureira et al., 2019; Gualdoni et al., 2021, 2022a).

Alcohol disrupts the molecular angiogenic network governing placental vascular development. Although angiogenesis and vascular remodeling are fundamental determinants of placental development, relatively few studies have examined the early molecular mechanisms responsible for alcohol-induced placental vasculopathy. Available evidence from human and murine experimental models indicates that maternal alcohol exposure can disrupt the molecular regulating complex mechanisms of placental angiogenesis rather than affecting a single angiogenic pathway (Radek et al., 2008; Jégou et al., 2012; Gundogan et al., 2008, 2010, 2013, 2015; Gualdoni et al., 2022b, 2023). Under physiological conditions, placental vascular development is orchestrated by the integrated actions of HIFs, VEGF signaling, NO, MMPs, and redox-sensitive pathways, which together regulate endothelial proliferation, trophoblast invasion, extracellular matrix remodeling, and spiral artery transformation. Maternal alcohol exposure disrupts this finely balanced angiogenic network, shifting physiological redox signaling toward oxidative stress and thereby compromising placental vascular development.

Oxidative stress appears to represent one of the earliest and most important mechanisms underlying alcohol-induced placental dysfunction. Both alcohol and its metabolite acetaldehyde may contribute to the pathogenesis of altered placental growth and function on the first trimester (Lui et al., 2014). The alcohol in placenta is primary detoxified by other placenta**l** systems (CYP2E1) different from the alcohol dehydrogenase (ADH) and aldehyde dehydrogenase (ALDH) enzymes (Gemma et al., 2007), because of their low affinity/activity in placental tissues (Heller and Burd, 2013). Consequently, gestational alcohol exposure induces oxidative stress, lipid peroxidation, protein nitration, DNA damage, mitochondrial dysfunction, and inflammatory activation within placental tissues (Kay et al., 2000; Gundogan et al., 2008, 2010, 2013, 2015). Although antioxidant systems are present in the placenta (Qanungo and Mukherjea, 2000), persistent oxidative stress markedly impairs trophoblast function and increases the risk of pregnancy loss (Gundogan et al., 2010). In agreement, we demonstrated in mouse that perigestational alcohol exposure up to early organogenesis induces oxidative stress, protein nitration, lipid peroxidation, and DNA damage in decidual-trophoblastic tissues, accompanied by placental increased HIF-1α expression and reduced NO bioavailability (Coll et al., 2018; Gualdoni et al., 2021).

These oxidative alterations directly can affect the VEGF-dependent angiogenic machinery. Ethanol appears to dysregulate VEGF signaling according to developmental stage, tissue, alcohol dose, and exposure pattern (Radek et al., 2008; Jégou et al., 2012; Haghighi Poodeh et al., 2012). Experimental studies in chick extraembryonic tissues demonstrated impaired vascular development associated with reduced VEGF and VEGF receptor expression after moderate or heavy alcohol exposure (Gu et al., 2001; Tufan and Satiroglu-Tufan, 2003). Similarly, ethanol suppresses angiogenic gene expression in yolk sac membranes through ROS-dependent mechanisms (Wang et al., 2016), whereas diminished placental vascular density following early prenatal alcohol exposure is accompanied by reduced placental KDR expression at term (Holbrook et al., 2019). Conversely, transient VEGF overexpression has also been reported during acute placental injury, likely representing a compensatory response to alcohol-induced hypoxia (Haghighi Poodeh et al., 2012). Likewise, hypoxic placentas exhibit increased release of the soluble VEGF antagonist sFlt-1, further contributing to endothelial dysfunction and impaired angiogenesis (Roberts and Escudero, 2012). Together, these findings indicate that alcohol primarily disrupts the physiological balance of VEGF signaling rather than simply altering VEGF abundance.

Because VEGF signaling is tightly coupled to nitric oxide synthesis and matrix remodeling, alcohol-induced alterations extend downstream to both the NO and MMP systems. Chronic binge-like alcohol exposure decreases eNOS expression in uterine artery endothelium during late gestation (Ramadoss et al., 2011), whereas other experimental models have reported increased eNOS activity following acute ethanol exposure in placental tissues or endothelial cells (Acevedo et al., 2001), suggesting that NO responses are highly context dependent. Nevertheless, either excessive or insufficient NO production ultimately compromises placental vascular tone, increases uteroplacental vasoconstriction, promotes tissue hypoxia, and impairs fetal oxygen and nutrient delivery (Lui et al., 2014; Gundogan et al., 2015; Lo et al., 2017; Holbrook et al., 2019; Ohira et al., 2019). In parallel, gestational alcohol exposure modifies MMP transcription and activity, thereby disrupting trophoblast migration, extracellular matrix degradation, and maternal spiral artery remodeling (Kay et al., 2000; Gundogan et al., 2008; Orzabal et al., 2019). Collectively, these observations support the concept that oxidative stress acts as the central upstream event linking alcohol metabolism to dysregulation of the HIF-VEGF-NO-MMP signaling network, ultimately leading to defective placental angiogenesis, impaired vascular remodeling, placental insufficiency, and fetal cardiovascular programming.

Maternal alcohol exposure should therefore be viewed as a disruptor of the physiological angiogenic network rather than of individual angiogenic factors. The transition from physiological redox signaling to oxidative stress destabilizes the coordinated HIF-VEGF-NO-MMP axis, impairing trophoblast invasion, endothelial remodeling, spiral artery transformation, and labyrinth vascularization. This integrated molecular disruption provides a mechanistic framework linking early placental vasculopathy with fetal cardiovascular programming through the placental-fetal cardiovascular axis.

## Mechanistic model linking abnormal placenta with fetal cardiovasculopathy programming after maternal alcohol exposure

Maternal alcohol consumption, even when restricted to the early peri-implantational period or early organogenesis, may initiate placental vascular abnormalities that persist throughout gestation and ultimately contribute to fetal cardiovascular programming. Although the precise cellular and molecular mechanisms remain incompletely understood, accumulating experimental evidence from murine models suggest that early alcohol exposure disrupts placental angiogenesis and trophoblast development, resulting in long-lasting defects *in utero*placental vascularization and fetal growth (Coll et al., 2018; Ventureira et al., 2019; Gualdoni et al., 2021, 2022a, 2022b, 2023). Based on these findings, we propose an integrated mechanistic model linking placental angiogenic dysfunction with fetal cardiovascular programming (**Figure 2**).

**Figure 2:**
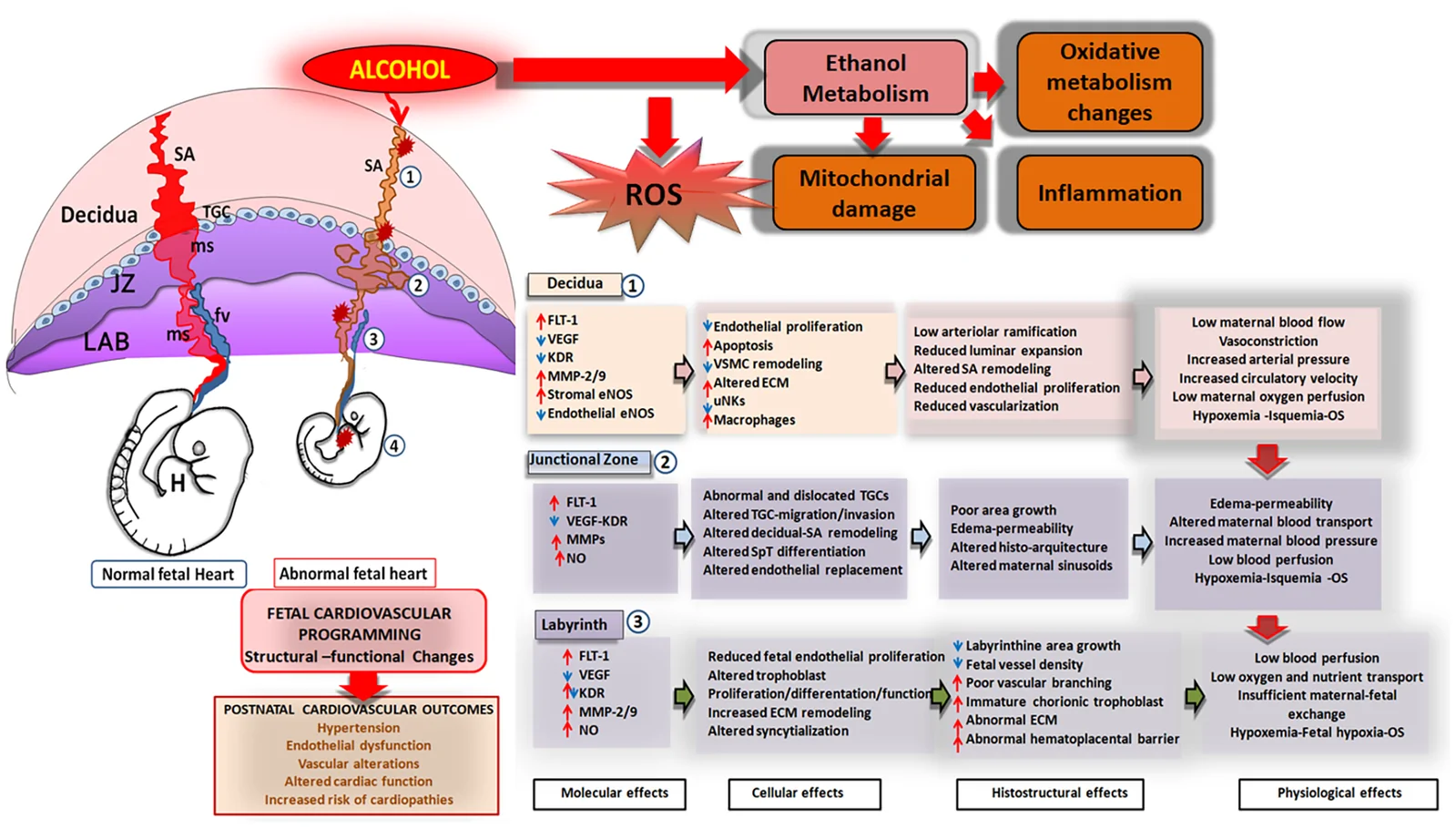
Mechanistic framework linking maternal alcohol-induced placental vascular dysfunction with fetal cardiovascular programming. Proposed sequential model illustrating how maternal alcohol exposure disrupts placental angiogenic homeostasis and programs long-term cardiovascular disease. Ethanol metabolism induces oxidative stress through reactive oxygen species (ROS) generation, mitochondrial dysfunction and HIF-1α activation, inducing inflammation and altering the oxidative metabolism. (1) Alcohol, ethanol metabolism-derived products and ROS first disrupt decidual angiogenesis, altering the maternal VEGF/KDR-MMP-NO signaling network, reducing uNK cells and spiral artery (SA) remodeling, reducing expansion and thereby altering physiological decidual vascular parameters. (2) By direct action of SA-circulating alcohol and decidual tissue-derived ROS, junctional angiogenic pathways are disrupted, compromising trophoblast proliferation, differentiation, migration and invasion, resulting in defective trophoblast-mediated SA remodeling and persistent abnormalities at the decidual-junctional interface. (3) Maternal vascular insufficiency and perfusion and direct circulating alcohol, VEGF-dependent signalings are disrupted, and consequently trophoblast differentiation and function and branching morphogenesis are impaired in the labyrinth. (4) With the establishment of altered structural and functional placenta, oxygen and nutrient exchange and molecular interchanges at the fetal-maternal interface are compromised. Impaired placenta-fetal cardiovascular axis may induce fetal restriction growth, hipoxia, altered fetoplacental hemodynamia, abnormal cardiac angiogenic factor expression and biomechanical signals, compromising cardiomyocyte proliferation, myocardial remodeling, coronary vascular development, septation and valvulogenesis, and ultimately influencing fetal cardiac development and promoting susceptibility to cardiovascular disease later in life. Lab, labyrinth; TGC, trophoblast giant cells; fv, fetal vessels; JZ, junctional zone; ms, maternal sinousoids.

In the first stage of placental disruption, alcohol arrives to maternal face via uterine artery, and impact to stromal, extracelular matrix and spiral arteries of decidual tissue (**Figure 2**, step 1). By the decidual ethanol metabolization, acetaldehyde, acetate, increased NADH/NAD and ROS are produced. As consequence of this maternal exposure, oxidative stress causes protein nitration, yielding changes in their expression and functions, lipid peroxidation, DNA damage, and oxidative metabolism imbalances by which probably lead to increase of lactate, triglycerides and cholesterol, decreased glycolysis, protein acetylations, mechanisms that may be related to changes in epigenetic programs. In addition, product of OS, mitochondrial damage may yield to dysfunction and leading to persistent tissue hypoxia and HIF-1α stabilization (Coll et al., 2018; Gualdoni et al., 2021). In addition, as consequence of OS, a proinflammatory state is generated in decidual tissue, which may produce increase of cytokines that further enhances the tissue damage. Although transient hypoxia normally stimulates physiological placental angiogenesis, sustained oxidative stress disrupts this adaptive response by altering the VEGF signaling system. Decreased VEGF-KDR signaling, together with increased expression of the anti-angiogenic receptor FLT-1, impairs downstream activation of eNOS and altering the nitric oxide bioavailability, the expression and activation of MMP-2 and MMP-9. These molecular effects conduce to several cellular defects, including reduced endothelial proliferation, altered extracellular matrix remodeling. Simultaneously, alcohol decreases uterine NK-cell abundance and function, further compromising decidual angiogenesis (Ventureira et al., 2019; Gualdoni et al., 2023). The resulting failure of spiral artery remodeling yield to poorly dilated, expanded and high-resistance maternal vessels, reducing uteroplacental perfusion and increasing local hipoxia in the maternal face of early placenta exposed to alcohol.

At trophoblast-decidual interface, abnormal decidual vascularization, low perfusion and oxygen biodisponibility, together with an maternal oxidative-proinflammatory environment, junctional trophoblast function and invasion may result affected (**Figure 2**, step 2). Thus, persistent hipoxia and oxidative stress impair the expression and activation of VEGF-dependent signalings, determining abnormal differentiation and function of trophoblast giant cells and spongiotrophoblasts, limiting trophoblast-mediated maternal vascular transformation and destabilizing the fetal-deicidual interface (Gualdoni et al., 2021). Consequently, structural and functional abnormalities develop within the junctional zone, further amplifying placental vascular insufficiency.

Defective labyrinthine angiogenesis and fetal placental circulation (**Figure 2**, step 3), may be also a consequence of altered maternal vascularization and deficient perfusion into this placental layer. Together with disrupted chorionic trophoblast differentiation in the labyrinth, because of changes in oxygenation conditions, both the development and branching of the labyrinth’s fetal vasculature is compromised. These effects may be originated from dysregulated VEGF/KDR-FLT1 signaling, altered eNOS activity, abnormal NO production and disturbed MMP expression that interfere with branching morphogenesis and maturation of the placental fetal vascular tree. The resulting reduction in labyrinthine vascular density limits fetal-maternal oxygen and nutrient exchange, leading to placental growth restriction, developmental abnormalities and persistent placental insufficiency (Gualdoni et al., 2021, 2022a; Coll et al., 2011, 2017).

These adverse effects and disruption of angiogenic mechanisms caused by maternal exposure to alcohol, even from early stages of gestation (gastrulation (GD7-8.5) and organogenesis (GD9-11)) on decidual and trophoblastic tissues (junctional zone and labyrinth) ultimately lead to the establishment of an abnormal placenta at short or long term. In this context, once the definitive fetoplacental circulation is established in the labyrinth (GD11.5), placental dysfunction directly influences embryofetal cardiac development (**Figure 2**, step 4) through two complementary mechanisms. First, abnormal placental vascularization modifies the fetal transfer of nutrient and oxygen, and placental-derived signaling molecules, including angiogenic factors (VEGF, soluble FLT-1), hormones, growth factors, cytokines, extracellular vesicles, microRNAs and metabolic mediators. Within the developing heart, these abnormal transfer of placental signals to fetus may alter angiogenic and morphogenic molecular pathways and its regulation in the developing fetal heart, affecting cardiomyocyte proliferation, coronary vascularization, disturbing ventricular remodeling, septation and valvulogenesis, and inducing epigenetic and metabolic reprogramming. Second, impaired placental vascularization alter fetal hemodynamic forces by increasing vascular resistance, blood pressure, flow, velocity and shear stress within the fetoplacental circulation.

In overall, these molecular and biomechanical signals constitute the functional coupling between the placenta and the fetal cardiovascular system and together amplify cardiac vulnerability during late gestation.Although many of these fetal cardiac responses initially represent adaptive mechanisms to an adverse intrauterine environment, they ultimately increase susceptibility to long-term cardiovascular dysfunction, including endothelial dysfunction, arterial stiffness, hypertension and other adult cardiovascular diseases.

Collectively, this proposed model places the placenta as the primary biological mediator through which maternal alcohol exposure is translated into fetal cardiovascular injury. Rather than representing independent pathological events, placental vasculopathy and fetal cardiogenesis appear to constitute sequential components of a common developmental pathway, where early disruption of placental angiogenic homeostasis initiates a cascade of molecular, cellular and hemodynamic events that culminate in lifelong cardiovascular programming.

## Concluding remarks and future perspectives

Maternal alcohol consumption during pregnancy represents a major developmental insult capable of perturbing the integrated placenta-embryofetal cardiovascular axis. The experimental evidence reviewed here indicates that prenatal alcohol exposure can disrupt placental angiogenesis, trophoblast differentiation and invasion, spiral artery remodeling, labyrinthine vascularization, and redox homeostasis, resulting in placental hypoxia, oxidative stress, and impaired maternofetal perfusion. These alterations may modify the molecular angiogenic, metabolic, endocrine, oxygen-dependent, and hemodynamic signals that support fetal cardiovascular development during critical developmental windows.

Collectively, current findings support the concept that alcohol-induced placental vasculopathy may represent an important mechanistic intermediary linking maternal alcohol exposure with fetal cardiovascular injury and long-term cardiovascular susceptibility. Within this emerging framework, placental dysfunction and fetal cardiogenesis are not considered independent processes, but components of an interconnected developmental system in which disruption of angiogenic and redox homeostasis, together with altered trophoblast function and fetoplacental hemodynamics, may converge to modify cardiovascular development and its subsequent trajectory. However, the relative contribution of direct embryofetal effects and indirect placental-mediated mechanisms remains incompletely defined, particularly across distinct developmental windows.

Several fundamental questions therefore remain unresolved. Future studies should establish the temporal sequence and reversibility of alcohol-induced placental vascular alterations, define the relative contribution of oxidative, inflammatory, metabolic, endocrine, and epigenetic mechanisms, and determine how placental-derived signals, including angiogenic factors, hormones, extracellular vesicles, microRNAs, and other mediators,interact with altered fetoplacental hemodynamics to influence fetal cardiac morphogenesis and maturation. Greater integration of placental phenotyping, fetal cardiovascular imaging, developmental cardiology, vascular physiology, and molecular and epigenetic approaches will be essential to distinguish causal mechanisms from adaptive responses and to strengthen the translational relevance of experimental findings.

Ultimately, this emerging paradigm positions the placenta not simply as a target of alcohol toxicity or a transient organ of gestation, but as a dynamic developmental interface through which adverse maternal exposures may be translated into persistent alterations in fetal cardiovascular development. A deeper understanding of the placental mechanisms underlying alcohol-associated cardiovascular programming may enable the identification of early biomarkers of fetal cardiovascular risk and reveal opportunities for preventive or therapeutic interventions aimed at preserving placental vascular homeostasis and reducing the lifelong burden of cardiovascular disease associated with prenatal alcohol exposure.
